# A sensory memory to preserve visual representations across eye movements

**DOI:** 10.1038/s41467-021-26756-0

**Published:** 2021-11-08

**Authors:** Amir Akbarian, Kelsey Clark, Behrad Noudoost, Neda Nategh

**Affiliations:** 1 Department of Ophthalmology and Visual Sciences, University of Utah, Salt Lake City, UT USA; 2 Department of Electrical and Computer Engineering, University of Utah, Salt Lake City, UT USA

**Keywords:** Sensory processing, Extrastriate cortex

## Abstract

Saccadic eye movements (saccades) disrupt the continuous flow of visual information, yet our perception of the visual world remains uninterrupted. Here we assess the representation of the visual scene across saccades from single-trial spike trains of extrastriate visual areas, using a combined electrophysiology and statistical modeling approach. Using a model-based decoder we generate a high temporal resolution readout of visual information, and identify the specific changes in neurons’ spatiotemporal sensitivity that underly an integrated perisaccadic representation of visual space. Our results show that by maintaining a memory of the visual scene, extrastriate neurons produce an uninterrupted representation of the visual world. Extrastriate neurons exhibit a late response enhancement close to the time of saccade onset, which preserves the latest pre-saccadic information until the post-saccadic flow of retinal information resumes. These results show how our brain exploits available information to maintain a representation of the scene while visual inputs are disrupted.

## Abstract (web summary)

A late enhancement of the perisaccadic neural response may exist in extrastriate areas. Here the authors show this preserves pre-saccadic information until the post-saccadic information is received, maintaining an integrated representation of the visual scene across saccadic eye movements.

## Introduction

About three times each second, saccadic eye movements (saccades) interrupt the flow of retinal information to higher visual areas^1–3^. To produce a stable sense of vision, our brain is believed to reconstruct at least some portion of the visual world during these gaps^4^. Studying the nature and source of information used by the visual system to fill the perceptual gap during saccades has been a central focus of psychophysicists, physiologists, and cognitive neuroscientists for decades^2,4–7^. The question holds a critical significance as it directly targets the constructive nature of visual perception: how a continuous perception of the visual scene emerges out of retinal input frequently disrupted by saccades. The prevailing idea is that prefrontal and parietal areas can provide neurons in visual areas with other sources of information beyond their receptive field (RF) to enable them to fill the gap during saccades^4,8,9^. For example, it has been shown that parietal and prefrontal neurons preemptively process information from their future receptive field (FF)^10–20^, and prefrontal neurons across visual space develop a target-centered representation by responding presaccadically to remote stimuli presented around the saccade target (ST)^21,22^. Despite these theories, a direct assessment of the nature of the information filling the transsaccadic gap in visual areas—in other words, the neural basis of transsaccadic integration—is still missing.

In order to understand the neural basis of transsaccadic integration, we recorded the activity of extrastriate neurons in area V4 and the middle temporal (MT) cortex of macaque monkeys, and developed a computational model to allow an instantaneous readout of the visuospatial representation from spiking responses on the timescale of a saccade. This decoding framework revealed that throughout saccades neural activity represented either the presaccadic or the postsaccadic visual scene, leaving no gap in the visual representation. More importantly, this approach allowed us to decompose the spatiotemporal sensitivity of individual neurons to trace the components required for this transsaccadic integration. This feature enabled us to identify a neural phenomenon as a key player for transsaccadic integration: extrastriate neurons exhibit a late enhancement of responses to stimuli appearing in the original RF around saccade onset, which preserves the history of the visual scene until the new retinal information arrives. This phenomenon, which was verified in both V4 and MT, reveals how by actively maintaining the presaccadic representation, extrastriate neurons can contribute to a stable uninterrupted perception of the visual scene during saccades.

## Results

### Tracing changes in the visual sensitivity of extrastriate neurons across saccades

**Fig. 1:**
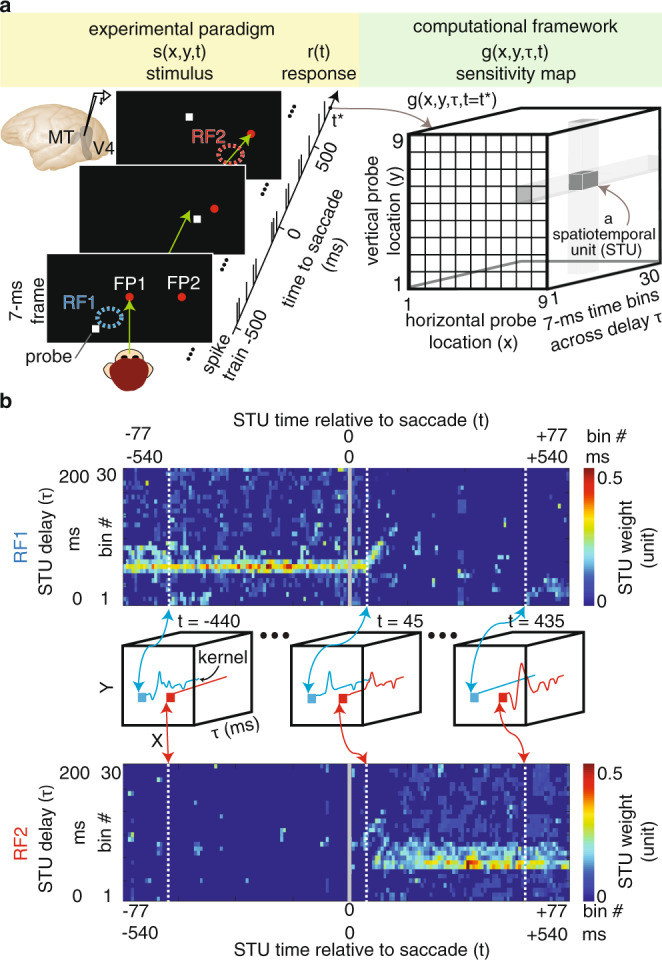
Decomposing sensitivity maps of neurons across saccades. **a** Experimental paradigm: monkey saccades from a fixation point (FP1) to a peripheral target (FP2). Throughout, visual probes (white squares) appear at pseudorandom locations. Green arrow shows gaze direction. Computational framework: composition of neuron’s sensitivity map across delays and locations, illustrated schematically for one time point relative to saccade. **b** A sample neuron’s estimated STU weights are shown for RF1 and RF2, across time to saccade and delay. The middle row shows example kernels (estimated as a weighted combination of STUs for RF1, blue, and RF2, red, for example pre-, peri- and post- saccadic time points).

We recorded the spiking activity of 291 V4 and 332 MT neurons while monkeys performed a visually guided saccade task (Fig. 1a, left, also see “Methods”, Supplementary Information section SOM1, and Supplementary Fig. 1). The animals maintained their gaze on a central fixation point (FP1) for 700–1100 ms and, upon the FP1 offset, shifted their gaze to a peripheral target (FP2) and fixated there for another 560–750 ms. Prior to, during, and after saccades, 7-ms duration small visual stimuli (probes) were presented pseudorandomly within a matrix of 9 × 9 locations covering the FP1, FP2, and the estimated receptive fields of the neurons before and after the saccade (RF1 and RF2). In most of the sessions (*n* = 85), the FP2 location was fixed across all trials. In some of the sessions (*n* = 23), the FP2 was randomly placed either on the right or left side of the FP1 (at the same radius; see details in Supplementary Information section SOM1-2). In order to assess how extrastriate neurons represent the visual world we needed to trace the dynamics of their sensitivity as it changes during saccades. The neuron’s sensitivity $(g)$ at a certain time relative to the saccade $(t)$ is defined as the efficacy of a stimulus at a certain location ($x$ and $y$) presented at a specific delay $(\tau )$ before that time to evoke a response in that neuron (Fig. 1a, right). In order to assess this sensitivity map, we employed a computational approach. First, we decomposed the time and location into discrete bins of ~3–6 degrees of visual angle (dva) and $7$ ms time bins (resolution of probes). For the duration and precision of our experimental paradigm, a full description of a neurons’ sensitivity required evaluation of ${10}^{7}$ of these spatiotemporal units (STUs). We used a dimensionality reduction algorithm to select only those STUs that contribute to the stimulus-response correspondence (see Methods; Supplementary Fig. 2). This unbiased approach excluded $\sim\! 99.9 \%$ of STUs, making it feasible to evaluate the contribution of the remaining $\sim \!\!{10}^{4}\,$ STUs to the response of the neuron (8899.17 ± 113.97 STUs per neuron). We developed a computational model to predict the neuron’s response based on an estimated sensitivity map. Using a gradient descent algorithm, we asked the model to determine the contribution (weight) of each STU of the sensitivity map, with the goal of maximizing the similarity between the model’s predicted response and the actual neuronal response (see “Methods” and Supplementary Information section SOM2). Figure 1b shows examples of weighted STUs for a model of an example MT neuron at a location inside its RF1 (top) and RF2 (bottom) for various times relative to the saccade. Note that the combination of STU weights across delays for a certain time and location is a representation of the neuron’s sensitivity, classically known as its “kernel” (middle panel). Overall, the model performed well in capturing the dynamics of neuronal responses, as well as providing high temporal resolution sensitivity maps of neurons (see “Methods” and Supplementary Information section SOM 2; Supplementary Figs. 3, 4)^23^.

### A model-based readout of transsaccadic integration

**Fig. 2:**
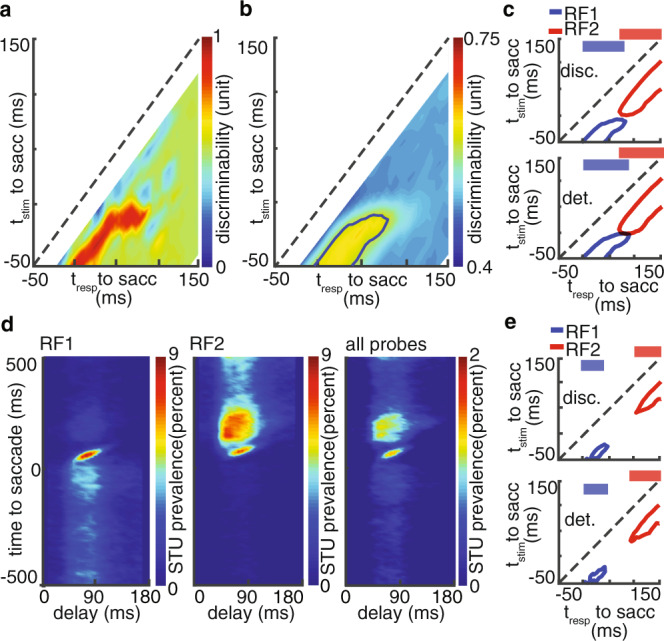
Quantifying the spatial integration and assessing the contributing perisaccadic modulatory components. **a** Location discriminability measured via model decoding for a sample MT neuron around its RF1. **b** Location discriminability around RF1 for the population of 623 neurons. The contour shows where the discriminability exceeds a threshold. **c** Temporal profiles of discriminability (top) and detectability (bottom) for RF1 and RF2; continuous sensitivity is reflected in the overlap in the projections on top. Contours indicate the times at which discriminability/detectability were above a certain threshold. **d** Temporal characteristics of modulated STUs. **e** Temporal profiles of discriminability (top) and detectability (bottom) for RF1 and RF2 after removing modulated STUs.

The goal of our combined electrophysiological and computational approach is to identify the neural components underlying transsaccadic integration. This requires translating the sensitivity map to a readout of the visual scene (employing the decoding aspect of the model), and then using this readout to assess the transsaccadic integration around the time of saccades. By capturing the essential computations of the neuron, the model can be used to generate predictions about any unseen sequence of visual stimuli. An example of how this can be used to generate a readout of the visual scene is shown in Fig. 2a. The model is used to predict responses to 9 probes around RF1 at various times relative to saccade onset. For a pair of probes, the spatial discrimination was then measured using the area under the curve (AUC) in the Receiver Operating Characteristic method based on the model-predicted responses (see “Methods”; Supplementary Fig. 5a–d). Location discriminability is assessed by the average AUC across all pairs of probes, and is plotted for a single neuron at various times of its response (*x*-axis) for probes presented at different times relative to saccade onset (*y*-axis). Figure 2b shows the same location discriminability map for the population of 623 modeled neurons, and the blue contour indicates the times at which the response can differentiate between probe locations above a certain threshold (AUC>0.55). The same contour is shown in Fig. 2c, top, along with the contour assessed with a similar method based on the location discriminability around RF2. Consistent with the subjective perception of a continuous visual scene, there is no time at which spatial sensitivity is lost during saccades, as indicated in the contact between the red and blue regions, and the overlap in their projections along the response time dimension (Fig. 2c, top, overlap = 23.23 ± 4.92 ms; see “Methods” and Supplementary Information section SOM3). The same phenomenon was also observed when assessing the detection performance of neurons (Fig. 2c, bottom, see “Methods”). Therefore, tracing the capacity of neurons to decode location information, the model predicts no gap between encoding information from the presaccadic scene and the postsaccadic one.

The approach revealed an important insight about what exactly happens to the visual scene representation around the time of a saccade. As shown in Fig. 2b, c, responses up to 50 ms after saccade onset show that neurons consistently kept their spatial sensitivity to stimuli as early as 50 ms before that response time (deviation from the line of unity, which is also a reflection of neuronal response latency). Interestingly, the blue curve is slightly farther from the line of unity around the time of the saccade (~74 ms deviation for response times of 50–90 ms), implying that the neuron loses its sensitivity to more recent stimuli and instead remains sensitive to stimuli presented earlier in time, a phenomenon which could contribute to filling the perceptual gap during saccades (see “Methods” and Supplementary Information section SOM4 for verification at the neuronal level; Supplementary Fig. 5e, f).

### Identifying the perisaccadic modulations required for transsaccadic integration

Having confirmed that the readout of the visual scene is indeed integrated across saccades, and with evidence that this integration is accompanied by a change in the temporal dynamics of the neuronal response, we started our search to identify the exact extrastriate mechanism underlying this phenomenon. Importantly, the model provides the ability to independently manipulate individual components of neuronal sensitivity and assess their impact on both individual neuronal responses and on the visuospatial representation. This ability proved to be a very powerful tool in identifying the basis of the continuous visual representation across saccades. First, we identified the times at which saccades alter extrastriate neurons’ sensitivity by identifying the STUs whose contribution changes during saccades compared to fixation (see Methods). The temporal distribution of saccade-modulated STUs is shown in Fig. 2d for RF1, RF2, and all locations, across all modeled neurons. On average $\sim \!\!26 \%$ of STUs were modulated during saccades (2342.89 ± 45.36). Nulling these modulated STUs in the model, i.e. replacing their weights with the fixation weights, resulted in a clear gap in neurons’ sensitivity to visual information. Unlike the intact model in Fig. 2c, the model lacking the perisaccadic modulations (Fig. 2e) not only did not show any overlap between RF1 and RF2 sensitivity, it even showed a gap in the visuospatial representation—a temporal window during which extrastriate neurons are not sensitive to stimuli at either location (overlap = −36.88 ± 6.18 ms; see “Methods” and Supplementary Information section SOM3, Supplementary Fig. 5g, h for V4 and MT neurons separately). Supplementary Fig. 6a, b shows the method for quantifying overlap and the effect of eliminating modulated STUs on overlap time for the population of individual neurons models. These results demonstrate the necessity of perisaccadic extrastriate changes for maintaining an integrated representation of visual space across saccades.

**Fig. 3:**
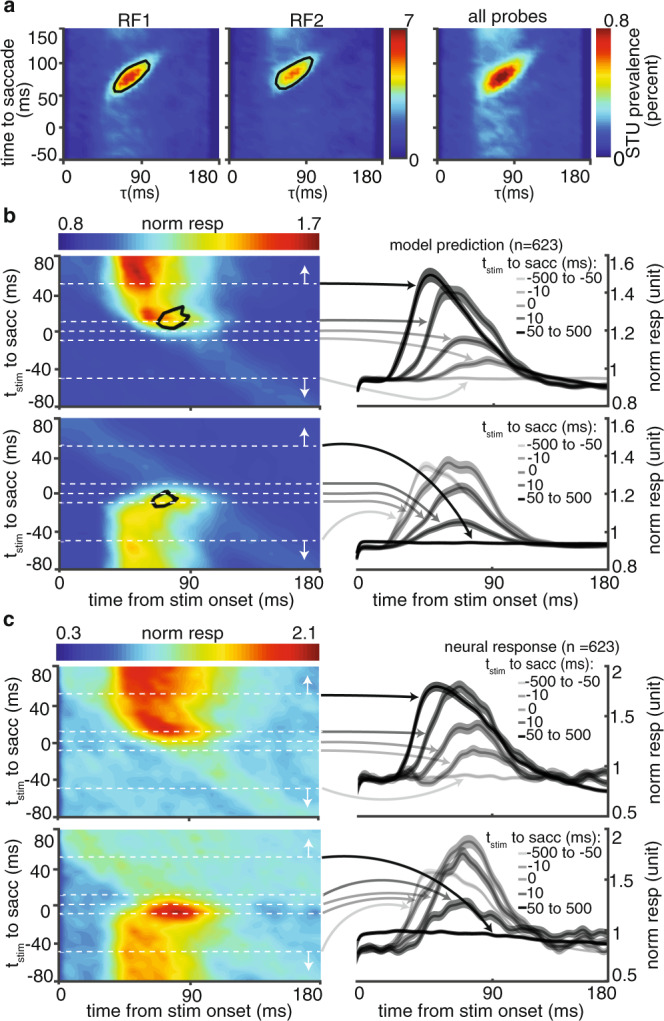
Identifying the neural basis for an integrated representation of visual space. **a** Temporal characteristics of integration-relevant STUs. Contours represent the times where the prevalence of integration-relevant STUs over the population of 623 neurons is greater than 50% of the maximum prevalence value. **b** Model-predicted response of the population across time to saccade and delay. The black contours in (**a**, **b**) indicate the response components driven by integration-relevant STUs (the same contours aligned to different times). Line traces on the right show the average population model-predicted firing rate (mean±SEM), at five sample times relative to the saccade (dashed white lines on the left). **c** Plots are as in (**b**), but with actual neuronal responses. Line traces on the right represent the population peristimulus time histogram (PSTH) at sample time points.

Numerous psychophysical phenomena happen during saccades: targets of eye movements are processed better, sensitivity to detect changes and displacements of other objects are reduced, and perception of time and space alters^24^. Thus maintaining an integrated representation of space is only one of multiple perisaccadic perceptual phenomena, and may only depend on a subset of perisaccadic changes in sensitivity. Therefore, while Fig. 2 verified the necessity of perisaccadic sensitivity changes for this integration, we still need to determine exactly which changes are specifically related to transsaccadic integration. We defined the integration, based on a model readout and then induce assumption-free alterations into the model to determine which of the modulated STUs (Fig. 2d) are essential for an integrated representation of space, i.e. altering the model readout from Fig. 2e to c (See “Methods”; Supplementary Fig. 6c). Nulling this integration-relevant subset of modulated STUs also results in a gap in the detectability and discriminability maps (Supplementary Fig. 6d), confirming that the search algorithm for extracting integration-relevant STUs from the saccade-modulated STUs successfully identifies the modulations required for transsaccadic integration. The unbiased search within the space of STUs revealed the times, delays, and locations of “integration-relevant STUs” (Fig. 3a) (17.04 ± 0.23% of the modulated STUs were integration relevant).

### Transsaccadic integration depends on the late enhancement of neural responses

Importantly, the model can then be used to link the integration-relevant STUs to specific components of the neural response. For example, the regions inside the black contours in Fig. 3a are the integration-relevant STUs for RF1 and RF2 locations for all modeled neurons, and the black contours in Fig. 3b highlight the stimulus-aligned response component generated by those specific integration-relevant STUs at RF1 and RF2. This approach isolated an alteration in the dynamics of responses to RF1 and RF2 stimuli presented within 10 ms of saccades as the neural substrate for an integrated representation of visual space. As indicated in the right panels, around the time of the saccade, the early part of the response to RF1 probes gradually disappears and a late response component emerges instead (which disappears after the eye has landed on the second fixation point). For RF2 probes, a late component emerges first and gradually earlier components add to it to form the stimulus-aligned response of the neuron during the second fixation. The same phenomena observed in the model were also seen in the response of the population of neurons (Fig. 3c). The phenomenon occurring at RF2 is reminiscent of the previously reported FF remapping (see Supplementary Information section SOM5; Supplementary Fig. 7)^11,13^. The elongation of the RF1 response (which we call ‘late response enhancement’), however, is an unanticipated finding and provides reassurance that our unbiased search is casting a wide net to identify the neural basis for an integrated representation of visual space.

**Fig. 4:**
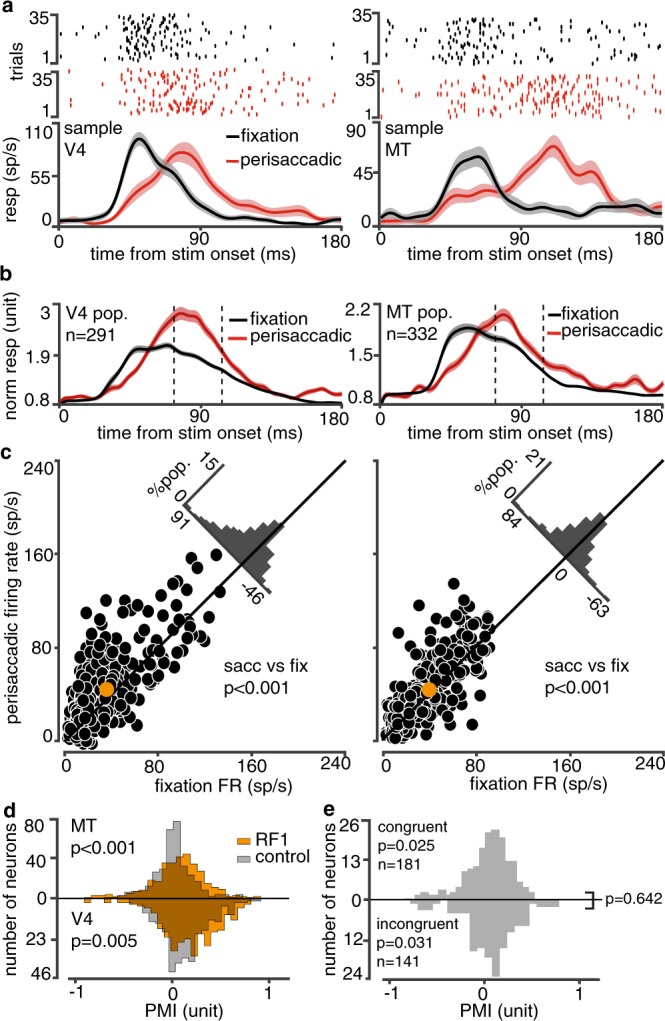
Late modulation of perisaccadic RF1 response. **a**, **b** Responses (mean ± SEM over trials of a single neuron, or across neurons) of sample neurons (**a**) and the neural population (**b**) to perisaccadic versus fixation RF1 probes in area V4 (left) and MT (right). Dashed lines indicate the late response window used in (**c**–**e**). **c** The average late response of V4 (left) and MT (right) neurons to perisaccadic versus fixation RF1 probes (V4, *p* = 9.10 × 10^−10^; MT, p = 3.31 × 10^−5^). Orange dots indicate center of mass. **d** PMI values for RF1 (orange) and control probes (gray), for the MT (top; *p* = 1.12 × 10^−4^) and V4 (bottom) populations. **e** PMI values for saccade directions congruent (top) versus incongruent (bottom) with MT neurons’ preferred motion direction. pop., population. ‘*n*’ indicates the total number of neurons with congruent or incongruent saccade directions (including data from single-direction and two-direction recording sessions). *P*-values above and below *y* = 0 in (**c**–**e**) are for two-sided Wilcoxon signed-rank tests (median of distribution vs. 0), and for a two-sided Wilcoxon ranked-sum test of congruent versus incongruent on the right in (**e**). Source data are provided as a Source Data file.

The phenomenon of late response enhancement was observed in populations of both V4 and MT neurons. Figure 4a shows the rastergram and the average response of sample V4 (left) and MT (right) neurons. The average response of these neurons during 75–105 ms after probe onset in V4 and 75–145 ms after probe onset in MT neurons increased by a factor of 2.31 and 2.80 for stimuli appearing around the saccade compared to fixation (V4_fixation_ = 25.09 ± 1.04 sp/s, V4_saccade_ = 58.06 ± 8.13 sp/s, p < 0.001; MT_fixation_ = 19.11 ± 0.62 sp/s, MT_saccade_ = 53.61 ± 5.40 sp/s, *p* < 0.001 Wilcoxon rank-sum test). As shown in Fig. 4b, c both V4 and MT populations exhibited an enhanced late response to their RF stimulus around the saccade compared to fixation (V4_fixation_ = 36.15 ± 1.61 sp/s, V4_saccade_ = 45.66 ± 1.89 sp/s, *p* < 0.001; MT_fixation_ = 40.97 ± 1.47 sp/s, MT_saccade_=45.39 ± 1.71 sp/s, *p* < 0.001) (see Supplementary Fig. 8a, b for sample neurons). This enhanced late response was accompanied by a suppression of early responses in both V4 and MT; we were predominately struck by the similarities between V4 and MT, both with respect to the prevalence and timecourse of the late response enhancement phenomenon (see Supplementary Information section SOM6; Supplementary Fig. 8). In order to examine the spatial selectivity of the observed phenomenon, for each probe we measured the perisaccadic modulation index (PMI) as the difference between saccade and fixation responses (75–105 ms after the stimulus) divided by their sum. PMI for RF probes was significantly greater than PMI for control probes outside the RF ($\varDelta {{{{{{\rm{PMI}}}}}}}_{{{{{{\rm{V}}}}}}4}=0.04\pm 0.01$, *p* = 0.005; $\varDelta {{{{{{\rm{PMI}}}}}}}_{{{{{{{\rm{MT}}}}}}}}=0.04\pm 0.01$, *p* < 0.001; Fig. 4d). We also confirmed that the late response enhancement phenomenon in MT is independent of whether the saccade direction is congruent or incongruent with the preferred motion direction of the neuron, ruling out saccade-induced retinal motion as the source of this phenomenon (PMI_congruent_ = 0.01 ± 0.01, *p* = 0.025; PMI_incongruent_ = 0.03 ± 0.02, *p* = 0.031, *p*_congruent vs. incongruent_ = 0.64, Wilcoxon rank-sum test; Fig. 4e; See “Methods” and Supplementary Information section SOM7; Supplementary Fig. 9). Thus, V4 and MT neurons display a delayed response to RF stimuli around the time of saccades, which is the feature of perisaccadic neural response modulation that the model identified as essential for integrating the visual representation across saccades.

## Discussion

Many studies have previously investigated changes in visual sensitivity around the time of saccades;^9,11,12,15,19–22,25–27^ and comprehensive reviews of those findings exist elsewhere^7,8^. To link these perisaccadic neurophysiological changes to perception, most of the studies have focused on either mechanistic models^28–33^, which try to reproduce the observed changes in perisaccadic neural responses, or computational models^24,34,35^, which try to provide a theoretical interpretation of changes in the neuron’s perisaccadic spatiotemporal dynamics that could account for perisaccadic perceptual stability. However, many of these studies focus on motor or attentional areas lacking strong visual selectivity, making it unclear how the reported perisaccadic changes in these areas can be translated into representational integration across saccades.

Here, we developed a data-driven, statistical framework integrated with electrophysiological experiments that enabled a quantitative description of the stimulus-response relationship on the fast timescale of a saccade. This quantitative description in turn allowed us to perform an unbiased search for the sensory signals in extrastriate areas that contribute to generating an integrated representation of visual space across a saccade. Indeed, assessing the sensitivity of visual neurons with high temporal precision and translating those sensitivity dynamics into their perceptual consequences were the two keys to identifying a neural correlate required for transsaccadic integration. Using a modeling approach to link changes in spatiotemporal sensitivity to visual perception, we found that extrastriate neurons are capable of ‘stitching’ their presaccadic representation to the postsaccadic one by maintaining a memory of the scene. Prior to a saccade, the response of extrastriate neurons at a certain time represents visuospatial phenomena occurring ~50 ms before. When the eye moves and the flow of retinal information is disrupted, instead of representing the visual events 50 ms ago, extrastriate neurons maintain a representation of events further back in time (~75 ms). This delayed response to the presaccadic scene, a brief ‘sensory memory’, prevents there being a period of time in which there is no visual information in the extrastriate representation. Thus, the computational approach not only allowed us to assess the visual representation with high temporal precision, it also enabled us to identify the exact neuronal response changes essential for creating an uninterrupted visual representation, revealing the phenomenon of late response enhancement, a sensory memory mechanism which can preserve information across saccades.

This discovery of late response enhancement dovetails with previous psychophysical studies suggesting the necessity of such a memory mechanism to preserve vision throughout the brief periods that the visual signal is lost during saccades or blinks^36,37^. Importantly, psychophysics experiments have implied that the preservation of vision across saccades might rely on mid- to high-level visual areas rather than on the earlier parts of the visual hierarchy^38,39^. Moreover, observing the late response enhancement phenomenon in both V4 and MT implies that this sensory memory is a characteristic of the visual system independent of whether the signal originates from chromatic/achromatic or motion sensitive pathways earlier in the visual stream^40^. However, what mechanism drives this late enhancement in the perisaccadic responses of extrastriate neurons remains unknown. The intrinsic signal within these areas (e.g. due to the abrupt change in the flow of visual information) might be enough to trigger an enhanced late response, but these areas also receive a copy of the motor command (e.g. via the tectopulvinar pathway to MT^41^) as well as motor preparatory and attentional signals (via direct projections from the Frontal Eye Field^42,43^). Considering that V4 is thought to receive the motor command through MT, but the dynamics of perisaccadic response changes in MT did not lead those in V4 (Supplementary Information section SOM6; Supplementary Fig. 8c), it seems the motor command is unlikely to be the source of modulation in V4, and that the role of top-down and intrinsic signals and their interactions are more promising candidates for future studies.

This paper sits at the intersection of two rich lines of research: statistical modeling of neural encoding and saccadic modulation of neurons’ visual responses. In the decades since the perisaccadic remapping of visual responses was first reported in LIP^11^, perisaccadic response mapping has been increasing in spatial coverage and temporal precision to provide a more complete picture of changes in spatial sensitivity and their dynamics^20,21^. A model-based approach for the mapping of RFs at the level of single trials and with high spatial and temporal precision around the time of saccades represents the next step in this progression. Our computational methods extend the classical GLM models, widely used for modeling neural responses^44^, including for mapping classical, time-independent receptive fields in various brain areas^45^, to a time-dependent RF estimation on the millisecond timescale. Previous changes in extrastriate visual responses around the time of saccades includes FF remapping in both V4 and MT^19,20,46^. We see evidence of similar FF remapping in our own data (SOM5; although our probes are briefer, 7 ms compared to 25 ms for Neupane et al., or on screen for >600 ms prior to the go cue for Yao et al.). The FF remapping phenomenon they report is similar to our late response enhancement in that probes are presented prior to the eye movement and responses occur after (e.g., ‘memory’ rather than ‘predictive’ remapping); however, our finding is specifically for the presaccadic RF location, reflecting the memory of the presaccadic scene. These previous studies used a sparser sampling over either the space or time dimensions, and either did not probe the RF location^19^, or excluded probes near the time of the saccade^20^, hence they did not observe the late response enhancement we see here. Thus a more precise and complete method for assessing a neuron’s spatiotemporal sensitivity revealed a previously unreported phenomenon.

As shown (Fig. 4 and Supplementary Fig. 8), the late response enhancement follows suppression of earlier responses. Multiple psychophysical phenomena are observed around the time of saccades, including spatial compression^47,48^, temporal compression^49^, and saccadic omission^50^. Although creating a readout model of each of these phenomena is beyond the scope of this paper, it is nevertheless tempting to speculate that the observed early response suppression could contribute to saccadic omission. However, the time window of the observed neural suppression appears narrower than that of perceptual saccadic omission, suggesting that other previously reported phenomena, including saccadic suppression^25,27,51^ and backward masking^52–54^, likely also contribute to saccadic omission.

It is imperative to emphasize that the phenomenon of perceptual stability, the subjective experience of a stable world during saccades, might require more than an integrated sensory representation. Perceptual stability has been shown to rely on working memory mechanisms^55,56^, and information outside a retinotopic framework might also be involved^21^ (see Supplementary Information section SOM8; Supplementary Fig. 10). The current results, however, show clearly that for a short period of time, retinotopic visual areas are capable of maintaining a brief sensory memory while the input is disrupted, a resource that could be employed by other areas and frames of reference to generate a stable, uninterrupted sense of vision.

## Methods

### Experimental paradigm and electrophysiological data recording

All experimental procedures complied with the National Institutes of Health Guide for the Care and Use of Laboratory Animals and the Society for Neuroscience Guidelines and Policies. The protocols for all experimental, surgical, and behavioral procedures were approved by Institutional Animal Care and Use Committees of the University of Utah. Animals were pair-housed when possible and had daily access to enrichment activities. During the recording days, they had controlled access to fluids, but food was available ad libitum.

Four male rhesus monkeys (monkeys B, P, E, and O; Macaca mulatta) were used in this study. Monkeys performed a visually guided saccade task during which task-irrelevant square stimuli flashed on the screen in pseudorandom order (Fig. 1a). The monkeys were trained to fixate on a fixation point (FP1; a central red dot) located in the center of the screen. After they fixated, a second target (FP2; a peripheral red dot) appeared 10-15 degrees away. Then, after a randomized time interval between 700 and 1100 ms (drawn from a uniform distribution), the fixation point disappeared, cuing the monkeys to make a saccade to FP2. After remaining fixated on the FP2 for 560–750 ms monkeys received a reward. During this procedure, a series of pseudorandomly located probe stimuli were presented on the screen in a 9 by 9 grid of possible locations. Each stimulus was a white square (full contrast), 0.5 by 0.5 degrees of visual angle (dva), against a black background. Each stimulus lasted for 7 ms and stimuli were presented consecutively without any overlap, such that at each time point there was only one stimulus on the screen. The locations of consecutive probe stimuli followed a pseudorandom order, called a condition. In each condition, a complete sequence of 81 probe stimuli was presented throughout the length of a trial. Conditions were designed to ensure that each probe location occurred at each time in the sequence with equal frequency across trials.

For each recording session, the grid of the possible locations of the probes was positioned such that it covered the estimated pre- and postsaccadic receptive fields (RFs) of the neurons under study, as well as the FP1 and FP2. The spatial extent of the probe grids varied from 24 to 48.79 (mean ± SD = 40.63 ± 5.93) dva horizontally, and from 16 to 48.79 (mean ± SD = 39.78 ± 7.81) dva vertically (Supplementary Fig. 1e). The (center-to-center) distance between two adjacent probe locations varied from 3 to 6.1 (mean ± SD = 5.07 ± 0.74) dva horizontally, and from 2 to 6.1 (mean ± SD = 4.97 ± 0.97) dva vertically. For the MT neurons, the motion direction preference was assessed using a full field Gabor paradigm before the saccade task. The monkey maintained fixation while a full field Gabor stimulus, moving in one of 8 directions, was displayed for 800 ms. In 23 out of 108 sessions, the ST was randomly located either on the right or left side of the FP (at the same radius; see more details in Supplementary Information section SOM1-2); in the rest of the sessions, the ST remained at the same location within a session.

Throughout the entire course of the experiment, the spiking activity of the neurons in areas V4 and MT was recorded using a 16-channel linear array electrode (V-probe, Plexon Inc., Dallas, TX, Central software v7.0.6 in Blackrock acquisition system and Cheetah v5.7.4 in Neuralynx acquisition systems) at a sampling rate of 32 KHz, and sorted offline using the Plexon offline spike sorter and Blackrock Offline Spike Sorter (BOSS) softwares. The eye position of the monkeys was monitored with an infrared optical eye-tracking system (EyeLink 1000 Plus Eye Tracker, SR Research Ltd., Ottawa, CA) with a resolution of <0.01 dva (based on manufacturer’s technical specifications), and a sampling frequency of 2 kHz. Stimulus presentation in the experiment was controlled using the MonkeyLogic toolbox^57^. In total, data were recorded from 332 MT and 291 V4 neurons during 108 recording sessions. See Supplementary Information section SOM1 for further details.

### RF estimation

The RF1 and RF2 locations used to calculate detectability and sensitivity in Fig. 2 refer to the probe locations that generated the maximum firing rate during the fixation period before and after the saccade, respectively. For each probe location, the probe-aligned responses are calculated by averaging the spike trains over repetitions of the probe before or after the saccade (greater than 100 ms before or after the saccade onset), from 0–200 ms following probe presentation, across all trials. The response is then smoothed using a Gaussian window of 5 ms full width at half maximum (FWHM).

### Dimensionality reduction for computing neuron’s time-varying sensitivity map

The fast, complex dynamics of changes in the neurons’ spatial sensitivity across a saccadic eye movement demand a high-dimensional representation of neurons’ spatiotemporal kernels in order to capture those perisaccadic dynamics. For any time relative to saccade onset the set of stimuli driving the response can be described in terms of their location ($X$ and $Y$) and the delay between the stimulus presentation and the response time ($\tau$). The goal is to determine this sensitivity map and trace its changes across time (Fig. 1b). In our experiment, for a 200 ms delay kernel across 1000 ms of response time, this space could be decomposed into ~10^7^ spatiotemporal units (STUs; Fig. 1a). Since the stimulus presentation resolution is 7 ms, we represent the variation of sensitivity across the time dimensions using a set of temporal basis functions, ${{{{{{\mathscr{B}}}}}}}_{i,j}(t,\tau )$, whose centers are separated by 7 ms across $\tau$ and $t$ dimensions (Eq. (1)). This way we down-sample the time into a sequence of binned STUs whose values can change every 7 ms.

1 $${{{{{{\mathscr{B}}}}}}}_{i,j}(t,\tau )={{{{{{\mathscr{U}}}}}}}_{i}(\tau ){{{{{{\mathscr{V}}}}}}}_{j}(t)$$

where ${{{{{{\mathscr{U}}}}}}}_{i}(\tau )$ and ${{{{{{\mathscr{V}}}}}}}_{j}(t)$ are chosen to be B-spline functions of order two. $\left\{{{{{{{\mathscr{U}}}}}}}_{i}(\tau )\right\}$ span over the delay variable $\tau$, representing a 200 ms-long kernel using a set of 33 knots uniformly spaced at $\left\{-13,-6,\ldots ,204,211\right\}$ ms (in total, 30 basis functions), and $\left\{{{{{{{\mathscr{V}}}}}}}_{j}(t)\right\}$ span over the time variable $t$, representing a 1081 ms-long kernel centered at the saccade onset using a set of 159 knots uniformly spaced at $\{-554,-547,\ldots ,545,552\}$ ms (in total, 156 basis functions).

This representation reduces the dimensionality of the spatiotemporal sensitivity map by about two orders of magnitude, however, it is still far beyond the practical dimensionality for a computationally robust estimation of the sensitivity values^23^ using an experimentally tractable amount of data. The short duration of saccade execution makes it infeasible to acquire a large number of data points from all spatial locations and times relative to saccade onset. To address this, we use a statistical approach to identify the STUs whose presence significantly contributes to the neuron’s response generation at a given time. Supplementary Fig. 2 shows this pruning procedure. For each STU we compare the distribution of its weights estimated by fitting a generalized linear model (GLM) on 100 subsets of randomly chosen spike trains (35% of total trials) versus the control distribution obtained using 100 subsets of shuffled trials in which the stimulus-response relationship was distorted. The conditional intensity function (CIF) of this GLM is defined as,

2 $${\lambda }_{t}=f\left(\mathop{\sum }\limits_{\tau =1}^{T}{s}_{i,j}\left(t-\tau \right).\kappa .{{{{{{\mathscr{B}}}}}}}_{i,j}\left(t,\tau \right)\right)$$

where ${\lambda }_{t}$ is the instantaneous firing rate of the neuron, ${s}_{i,j}$ is the stimulus history of length $T$ at location $(i,j)$, $\kappa$ is the weight of a single STU, represented by basis function$\,{{{{{{\mathscr{B}}}}}}}_{i,j}(t,\tau )$, whose contribution significance is evaluated. An STU is discarded if the mean of these two weight distributions fails to satisfy the following condition:

3 $$\left|\mu -\widetilde{\mu }\right|\ge 1.5\widetilde{\sigma }$$

where $\widetilde{\mu }$ and $\widetilde{\sigma }$ are respectively the mean and standard deviation of the control weights distribution, and $\mu$ is the mean of the original weights distribution. This pruning process reduces the dimensionality of STU space to ~10^4^, which makes fitting of our encoding model to the sparse perisaccadic spiking data feasible and prevents an overfitted result. We then use only this subset of STUs to parameterize the linear filtering stage of an encoding model in a much lower dimensional space in order to determine the weights with which these STUs are combined to generate the neuron’s spatiotemporal sensitivity (Fig. 1b); at each time point relative to the saccade onset, the weighted combination of these STUs over probe locations and delay times describes the neuron’s sensitivity kernels (Fig. 1b, middle panel) defined as,

4 $${k}_{x,y}(t,\tau )=\mathop{\sum}\limits_{i,j}{\kappa }_{x,y,i,j}.{{{{{{\mathscr{B}}}}}}}_{i,j}(t,\tau )$$

where $\left\{{\kappa }_{x,y,i,j}\right\}$ are the weights of the STUs obtained by estimating the encoding model (defined in Eq. (5) below). Note that the summation is over the subset of ${{{{{{\mathscr{B}}}}}}}_{i,j}(t,\tau )$ whose corresponding STU was evaluated as significant according to Eq. (3), while the weights for the remaining STUs were set to zero.

This low-dimensional set of the selected STUs enabled us to fit our encoding model to the sparse perisaccadic data and characterize the encoding principles of each neuron at each time relative to the saccade.

### Encoding model framework and estimation

Models based on the GLM framework have been widely used to describe the neural response dynamics in various brain areas^44,58–61^, including the response dynamics induced by a saccade^62–64^. By regressing the neural response on the stimulus variables, GLM-based models have also been used for mapping the neurons’ RF, including the perisaccadic RFs in sensory^46,51^, or prefrontal^65,66^ areas. Our lab has recently developed a variant of the GLM framework, termed the sparse-variable GLM (S-model^23^, Supplementary Fig. 3), applicable to sparse spiking data, which tracks the fast and high-dimensional dynamics of information encoding with high temporal precision and accuracy. The S-model enables us to represent the high-dimensional and time-dependent spatiotemporal sensitivity of neurons using a sparse set of STUs selected through a dimensionality reduction process and estimate their quantitative contribution to spike generation on a millisecond timescale across a saccade. Using this set of STUs we parameterize the stimulus kernels ${k}_{x,y}(t,\tau )$ in the CIF of the S-model defined as

5 $${\lambda }^{\left(l\right)}(t)={{{{{\rm{f}}}}}}\left(\mathop{\sum}\limits_{x,y,\tau }{k}_{x,y}(t,\tau ){s}_{x,y}^{\left(l\right)}(t-\tau )+\mathop{\sum}\limits_{\tau }h(\tau ){r}^{\left(l\right)}(t-\tau )+b(t)+{b}_{0}\right)$$

where ${\lambda }^{\left(l\right)}(t)$ represents the instantaneous firing rate of the neuron at time $t$ in trial $l$, ${s}_{x,y}^{\left(l\right)}({{{{{\rm{t}}}}}})\in \left\{0,1\right\}$ denotes a sequence of probe stimuli presented on the screen at probe location $\left(x,y\right)$ in trial $l$ with 0 and 1 representing respectively an off and on probe condition, ${r}^{\left(l\right)}({{{{{\rm{t}}}}}})\in \left\{0,1\right\}$ indicates the spiking response of the neuron for that trial and time, ${k}_{x,y}(t,\tau )$ represents the stimulus kernel at probe location $\left(x,y\right)$, $h({{{{{\rm{\tau }}}}}})$ is the post-spike kernel applied to the spike history which can capture the response refractoriness, $b({{{{{\rm{t}}}}}})$ is the offset kernel which represents the saccade-induced changes in the baseline activity, ${b}_{0}={{{{{{\rm{f}}}}}}}^{-1}\left({r}_{0}\right)$ with ${r}_{0}$ defined as the measured mean firing rate (spikes per second) across all trials in the experimental session, and finally,

6 $${{{{{\rm{f}}}}}}\left(u\right)=\frac{{r}_{{\max }}}{1+{e}^{-u}}$$

is a static sigmoidal function representing the response nonlinear properties where ${r}_{{\max }}$ indicates the maximum firing rate of the neuron obtained empirically from the experimental data. The fitted models were successful in describing the dynamics of the recorded neural data (Supplementary Fig. 4). This choice of the neuron’s nonlinearity is consistent with an empirical nonlinearity estimated nonparametrically from the data. All trials are saccade aligned, i.e., $t=0$ refers to the time of saccade onset. Then using an optimization procedure in the point process maximum likelihood estimation framework, we fit the model to sparse spiking data at the level of single trials. The resulting encoding framework enables us to decipher the nature of saccade-induced modulatory computations in a precise and computationally tractable manner using the time-varying kernels representing the neuron’s dynamic sensitivity across different delays and locations for any specific time relative to the saccade.

### Details of discriminability and detectability analysis

The decoding aspect of the model enables us to develop a readout of the visual scene using the model-predicted responses. The model readout provides a detailed description of the neural decoding capability across a saccadic eye movement, which can be used to trace a specific perceptual phenomenon (in our case, visuospatial integration across saccades) and test the specific components of the neural response that the phenomenon relies on. By capturing the essential computations of the neuron, the model can be used to generate predictions about arbitrary sequences of visual stimuli not present in the experimental data. We have used this aspect of the model to predict how the decoding capability of the neural response changes across a saccade, in terms of its ability to detect the presence of a particular probe. The detectability of an arbitrary probe is measured by evaluating the ability to detect the presence of that particular probe from the model-predicted response; i.e., when that probe is presented (ON) versus when it is not (OFF). The detectability of probes can differ based on the time between the probe presentation and the time of response that the decoding is being based on (referred to as the delay). At any time in the neural response (denoted as $t$ in Supplementary Fig. 5a), the probe is only detectable if it is presented within a certain delay range ($\tau$; we evaluated delay values from 0-200 ms). During the fixation period, long before the saccade onset, the RF1 probe’s detectability is maximum around the latency of the neuron. However, during the perisaccadic period, the neuron becomes sensitive to different probes and with different latencies. Supplementary Fig. 5a shows how the detectability of the RF1 probe of a sample neuron (RF1 probe: ${s}^{\ast }$) is computed at an arbitrary time (${t}^{\ast }$) where the probe is presented at (${t}^{\ast }-{\tau }^{\ast }$). To measure the detectability of ${s}^{\ast }$ at ${t}^{\ast },{\tau }^{\ast }$ the AUC measure^67^ has been used to evaluate the difference between the distribution of responses evoked at ${t}^{\ast }$ (${\lambda }_{{t}^{\ast }}$) by the presence of ${s}^{\ast }$ at ${t}^{\ast }-{\tau }^{\ast }$ versus in the absence of ${s}^{\ast }$, each embedded within a 200 ms random sequence of other probes. The model’s predicted response at time ${t}^{\ast }$ (denoted as ${\lambda }_{{t}^{\ast }}$) is generated for 100 random sequences of probes, when specific probe ${s}^{\ast }$ is ON and 100 random sequences in which that probe is OFF, ${\tau }^{\ast }$ before ${t}^{\ast }$ (i.e., at time ${t}^{\ast }-{\tau }^{\ast }$). The detectability is then measured as AUC of the evoked response (${\lambda }_{{t}^{\ast }}$) to the ON versus OFF trials (histograms shown in Supplementary Fig. 5a). To calculate the average detectability, mean AUC was calculated across 20 repetitions for each time and delay combination, each repetition over a randomly selected 80% of ON and OFF trials. Supplementary Fig. 5b shows the detectability at a sample time ${t}^{\ast }=+10$ ms relative to the saccade where the RF1 probe is presented 140 to 20 ms before saccade onset ($-150 \, < \, \tau \, < -\kern-2.5pt 30\,{{{{{{\rm{ms}}}}}}}$); at this time, the RF1 probe is detectable only when it was presented around 55 ms before the response (normal latency of the neuron). To track detectability across times and delays, the detectability of probes is measured at different values of $t$ relative to the saccade and $\tau$ relative to each response time ($t$: 50 ms before to 300 ms after saccade with 10 ms steps, and $\tau$: 190 to 30 ms before response time with 10 ms steps, Supplementary Fig. 5c). The detectability map of the neuron for each probe location provides a quantification of the decoding capacity of the neuron across the eye movement (Supplementary Fig. 5c shows the map for the RF1 probe of a sample neuron); the shift in detectability from RF1 to RF2 is shown in Supplementary Fig. 5d. Over time, the probe location with the highest detectability shifts from RF1 to RF2, as shown for the population of neurons in Supplementary Fig. 5d; the contours show the times and delays at which one can detect the presence of the stimulus based on the response of the neuron, i.e., the AUC values are above a threshold of 0.61.

In a similar way, we used the decoding approach to measure the location discriminability of the neural response, in terms of ability to discriminate a probe from the immediately surrounding probes using the model-predicted response. To measure the location discriminability at each probe location, 100 random sequences were presented to the model with the center probe presented at a specific delay, and AUC was measured versus 100 trials where one of the adjacent probes was presented at the same delay. Mean sensitivity for each of the surrounding probes was then calculated across 20 AUC measurements, each using 80% of trials. The location discriminability reported in Fig. 2a–c and e are then the average of discriminability over 8 surrounding probes around the RF1 or RF2 probe. The thresholds used in Fig. 2b, c, and e are ROC > 0.57 for discriminability and ROC > 0.61 for detectability.

### Identifying modulated and integration-relevant STUs

As discussed previously, only the STUs at specific times and delays are contributing to the neural response generation (green STU in Supplementary Fig. 2a). When the spatial and temporal sensitivity of a neuron changes during the perisaccadic period, the distribution of STUs (across times and delays) will be altered. We defined modulated STUs as those for which the prevalence of STUs in a 3x3 window around that STU’s time and delay is significantly different for stimuli presented perisaccadically vs. during fixation. Each STU is considered modulated if the prevalence of STUs fulfills the following condition:

7 $$\sqrt{{{{{{\rm{|}}}}}}p({\tau }_{n},{t}_{m})-{p}_{1}({\tau }_{n}){{{{{\rm{|}}}}}}.{{{{{\rm{|}}}}}}p({\tau }_{n},{t}_{m})-{p}_{2}({\tau }_{n}){{{{{\rm{|}}}}}}} \, > \, h$$

where $p\left({\tau }_{n},{t}_{m}\right)$ is prevalence of STUs in a 3x3 window around the $n$th bin of delay and $m$th bin in time 1 < *n* < 30,1 < *m* < 156, ${p}_{1}\left({\tau }_{n}\right)\,$is the prevalence of the STUs across fixation period before saccade calculated over bins 1 to 60 spanning 540 to 120 ms before saccade onset at$\,n$th bin of delay, $1 \, < \, n \, < \,30$, and ${p}_{2}\left({\tau }_{n}\right)$ is the prevalence of STUs in the fixation period after saccade calculated over bins 120 to 156 spanning 280 to 540 ms after saccade onset at$\,n$th bin of delay, $1 \, < \,n\, < \, 30$, and $h$ is a significance threshold between 0 and 1. The threshold is set to value $h=0.7$ for illustration purposes in Fig. 2d. For analysis, the threshold value was set to $h=0.3$ in order to include all perisaccadic STUs that might play a role in transsaccadic integration.

As shown in Fig. 2c, the modulated subset of STUs (Fig. 2d), representing the STUs which are significantly different between the fixation and perisaccadic periods, play a major role in maintaining visuospatial integrity across the saccade. As shown in Fig. 2e, replacing the weights of the modulated STUs in the model with their fixation values results in a gap in the readout of the neural responses—and interruption in the detectability and discriminability at RF1 or RF2 in the perisaccadic period.

In the next step, a subset of modulated STUs is identified as contributing to this visuospatial integration—e.g., the continuity of transitioning sensitivity from RF1 to RF2 across the saccade (termed ‘integration-relevant STUs’). The contribution of each modulated STU to the transsaccadic integrity is quantified by evaluating its role in maintaining the sensitivity of the neuron to either the RF1 or RF2 location across a saccadic eye movement, by removing each modulated STU one at a time and testing whether the neuron’s sensitivity decreases. The stimulus kernels of the fitted models (${K}_{x,y}\left(t,\tau \right)$, Supplementary Fig. 3b, g) reflect changes in the neurons’ spatiotemporal sensitivity at each probe location ($x,y$) and delay ($\tau$) across different times to the saccade ($t$). The average spatial sensitivity of the neuron to the stimulus in RF1, ${h}_{1}(t)$, is quantified as:

8 $${h}_{1}(t)=\frac{{\sum }_{\left(x,y\right)\epsilon R{F}_{1}}{\sum }_{\tau =1}^{T}\left|{K}_{x,y}(t,\tau )\right|\,}{9\ast T}$$

where $1 \, < \, \tau\, < \, T$ is the delay parameter in the kernels (in this study $T=200$ ms as length of stimulus kernels), regarding the history of stimulus from time $t$, and $(x,y)$ are the nine probe locations around center of RF1 (Supplementary Fig. 5a). The spatial sensitivity index of RF1, ${h}_{1}(t)$, representing the average sensitivity in terms of the average absolute kernel values, drops after a saccade, while the spatial sensitivity index ${h}_{2}(t)$ for RF2 increases. A shared sensitivity index ($\delta$) across RF1 and RF2 is then defined as the minimum sensitivity to either location, measured across time relative to the saccade (gray area in Supplementary Fig. 6c). The shared sensitivity is defined as $\delta ={\sum }_{t=-500}^{+500}{{{{{\rm{min }}}}}}({h}_{1}(t),{h}_{2}(t))$ and each modulated STU is considered integration-relevant if nulling its weight results in a decrease in the shared sensitivity of the neuron to the RF1 or RF2.

### Reporting summary

Further information on research design is available in the Nature Research Reporting Summary linked to this article.

## Supplementary information

Supplementary Information

Reporting Summary

## Data Availability

The data supporting the findings of this study are available from the corresponding authors upon request. Sample neurons from the data generated in this study have been deposited in the GitHub page here: https://github.com/nnategh/SVGLM/tree/master/assets/data. Source data are provided with this paper.

## Source data

Source Data

## Code availability

Computational source codes for the model fitting are available at https://github.com/nnategh/SVGLM.
