# Supplementary material for: A sensory memory to preserve visual representations across eye movements
Source: Nat Commun. 2021 Nov 8;12:6449. doi: 10.1038/s41467-021-26756-0 (PMC8575989; doi:10.1038/s41467-021-26756-0)
Supplement: Supplementary file 1 — Supplementary Information [file 41467_2021_26756_MOESM1_ESM.pdf]

## Supplementary Information:

# A Sensory Memory to Preserve Visual Representations Across Eye Movements


Amir Akbarian[1], Kelsey Clark[1], Behrad Noudoost[1*], Neda Nategh[1,2*]

[1]Department of Ophthalmology and Visual Sciences, University of Utah, Salt Lake City, Utah, United States of America.
[2]Department of Electrical and Computer Engineering, University of Utah, Salt Lake City, Utah, United States of America.

*Correspondence to: neda.nategh@utah.edu, behrad.noudoost@utah.edu


### Contents


SOM 1) Experimental setup------------------------------------------------------------------------ 1

SOM 1-1) Animal preparation and surgical procedures ---------------------------------- 1

SOM 1-2) Data acquisition ------------------------------------------------------------------ 1

SOM 1-3) Location of RF centers ---------------------------------------------------------- 3

SOM 2) Encoding model framework and estimation ------------------------------------------- 4

SOM 2-1) Model fitting ---------------------------------------------------------------------- 4

SOM 2-2) Model evaluation ----------------------------------------------------------------- 6

SOM 3) Calculating the duration of overlap between RF1 and RF2 discriminability ------- 8

SOM 4) Detectability and discriminability in recorded neural data --------------------------- 9

SOM 5) Future field remapping ----------------------------------------------------------------10

SOM 6) Timing of the perisaccadic modulation in areas MT and V4 -----------------------12

SOM 7) Perisaccadic modulation of response in congruent versus incongruent saccades - 16

SOM 8) Saccade target remapping--------------------------------------------------------------17

Supplementary Figures 1-10 ------------------------------------------------------------------20

## SOM 1) Experimental setup

### 1-1) Animal preparation and surgical procedures

For each animal, a head-post was implanted on the skull using dental acrylic and orthopedic titanium screws. The surgical procedures were performed under strict aseptic conditions while the animals were anesthetized by Isoflurane. Recording chambers were mounted on the skull and fastened by screws and dental acrylic. The craniotomy was performed within the chamber, giving access to extrastriate visual areas including V4 and MT.

### 1-2) Data acquisition

The spike sorter program was employed to perform a principal component analysis, and clusters of spikes with similar waveform properties were manually classified as belonging to a single neuron (single unit). The sorted spikes were then read into Matlab to verify the presence of a visually-sensitive RF. From a population of 709 well-isolated neurons, 86 neurons were discarded because they did not respond to any probe stimuli before and/or after the saccade, and the rest were used for analyses. Visual stimuli were presented on a 24-inch ASUS VG248QE LED monitor with a resolution of 1920x1080 pixels with a refresh rate of 144 Hz, positioned 24-30 cm in front of the animal's eyes. A photodiode (OSRAM Opto Semiconductors, Sunnyvale CA), mounted on the lower left corner of the monitor, was used to record the actual onset and offset times of stimuli appearing on the screen with a continuous signal sampled and stored at 32 KHz.

Supplementary figure 1a illustrates the eye position, visual probe locations, and photodiode voltage in a sample trial. As shown in the figure, a separate stimulus recorded by the photodiode is toggled (on/off) at the onset of each visual probe. Supplementary figure 1b

illustrates the monitor's luminance decay profile measured as the average timecourse of the photodiode signal across all photodiode offsets in a sample session, indicating that luminance has completely disappeared in under 5 ms from visual probe offset. The fixation window around the FP was ±2 dva, and the fixation window following the saccade was ±4 dva from the ST. Supplementary figure 1c shows the distribution of saccade endpoint scatter across 108 sessions (population mean 0.62±0.02 dva). For each neuron, the saccade endpoint scatter was measured as the standard error of the distances between each saccade endpoint and the average endpoint across all saccades. Supplementary figure 1d illustrates the monkeys' reaction times across 108 sessions (217.48±3.24 ms).

In total, data were recorded from 332 MT and 291 V4 neurons during 108 recording sessions. Only one area (V4 or MT) was recorded in a given session. The saccade target location and saccade direction could vary from session to session. The various saccade target locations are shown in Supplementary figure 1e. In 23 sessions (in monkeys E and O), the monkey made saccades in two opposite horizontal directions, and the ST was randomly presented either right or left of FP (at the same radius) across trials (these data were used to test the effect of saccade direction, Supplementary Fig. 9). In the remaining 85 sessions, the ST was presented at only one location each day. In total, for 439 out of 623 neurons saccades were made to the left, and to the right for 146 out of 623 neurons, and in the remaining 38 neurons the saccades were made in other directions (195, 200, and 225 degrees). In the sessions where the monkey made saccades in two directions, the monkey performed more than twice as many trials as a single-direction session to accommodate enough trials for further analysis.

Prior to data collection, areas V4 and MT were identified based on anatomical landmarks and the pattern of neural responses to different stimuli (controlled with custom Matlab codes using the MonkeyLogic toolbox[1]). Before each session of recording, the boundaries of the RFs of the neurons were roughly mapped based on audible neural responses to a moving or stationary white bar on a black background at different directions and locations on the screen. For each session, the position of the grid on the screen and the distance between adjacent probes were adjusted to cover the estimated RFs and post-saccadic RFs of all channels, as well as the FP and ST. The positioning of the probe grids and saccade targets across all sessions is shown in Supplementary figure 1e.

Saccade endpoint variation was 0.62±0.02 dva across 108 sessions. Saccades were extracted based on the displacement between eye positions over a sliding time window and also the speed of such displacements. First, the eye positions (sampled at 2 kHz) were smoothed with a gaussian window of 5 ms full width at half maximum. Second, the eye position is calculated as the median of eye positions in a 50 ms window around each time point. Then for each time point, the eye displacements and speeds are calculated over a 10 ms window centered on that time point. A change in the eye position is considered a saccade if the displacement is greater than 6 dva and the speed of the displacement stays above 80 dva/s for 3 ms. Saccade onset was then defined as the first time when the eye speed reaches 80 dva/s.

**1-3) Location of RF centers**

The locations of RF centers for all recorded neurons are illustrated in Supplementary figure 1f. RF center calculations are based on the mean probe-aligned responses, averaged over a window of 25 ms around the time of maximum response for each neuron. The map is

then interpolated (interleaving 1000 points between each probe location) and thresholded at 0.99 of the maximum response. The center of RF is then calculated as the center of mass of the resulting spatial map. For the population of neurons, the average RF center was located 8.35±0.16 dva away from the initial FP (Supplementary Fig. 1f).

## SOM 2) Encoding model framework and estimation

Using the sparse variable generalized linear model (S-model) encoding model, we can capture the neuron's high-resolution spatiotemporal sensitivity using limited perisaccadic data. Using dimensionality reduction to identify the subset of STUs representing the stimulus-response relationship (see Methods) is a key step enabling us to successfully fit the S-model parameters using the sparse neuronal data during saccades. Then using an optimization procedure in the point process maximum likelihood estimation framework, we fit the model to sparse spiking data at the level of single trials. The resulting encoding framework enables us to decipher the nature of saccade-induced modulatory computations in a precise and computationally tractable manner using the time-varying kernels representing the neuron's dynamic sensitivity across different delays and locations for any specific time relative to the saccade.

### SOM 2-1) Model fitting

We model the spiking response using a conditionally inhomogeneous Poisson point process, with its CIF defined in Eq. 5 in the Methods. The probability of a spike train associated with this Poisson process is given by,

$$p\left(\boldsymbol{r}^{(l)}\middle|\boldsymbol{s}\right) = \prod_t p\left(r^{(l)}(t)\middle|\boldsymbol{s}\right) \propto \prod_t \left(\lambda^{(l)}(t)\Delta\right)^{r^{(l)}[t]} e^{-\lambda^{(l)}(t)\Delta} \tag{9}$$

where $\boldsymbol{s}$ is the sequence of input stimuli, and $\boldsymbol{r}^{(l)} = \{r^{(l)}(t)\}$ represents the sequence of binned spike counts with bins of size Δ ms on trial $l$. Here, the bin size was chosen equal to 1 ms which ensures that at most one spike can fall in each time bin. The point process log-likelihood ($LL$) of the observed spike trains given the model is as follows,

$$LL(\boldsymbol{\theta}) = \sum_{l,t}\left(r^{(l)}(t).\log\left(\lambda^{(l)}(t).\Delta\right) - \lambda^{(l)}(t).\Delta\right) \quad (10)$$

where $\boldsymbol{\theta}$ denotes the set of parameters used to describe the model kernels defined in Eq. 5 in the Methods. Note that $\kappa_{x,y,i,j}$, representing the weights of STUs in the stimulus kernels, is among this set of parameters**.** The parameters in $\boldsymbol{\theta}$ are estimated by maximizing the log-likelihood function in Eq. 10. The sigmoidal nonlinearity in the S-model's CIF (Eq. 5 in the Methods) makes the $LL$ function not convex, meaning it may not give a unique optimal solution (more details in[2, 3]). Also, considering the number of data points (spiking events) relative to the number of model parameters to be estimated, this optimization may be subject to the overfitting problem. To handle these nonconvexity and overfitting challenges and find a robust and interpretable solution among other possible solutions, we adopted several parameter selection, sparsity, and smoothness regularization strategies. These strategies included early stopping, representation of model kernels using smooth basis functions, data-informed initialization, an iterative, robust gradient-based search algorithm, and cross validation, in addition to those strategies described in the dimensionality reduction section (see Methods) for the stimulus kernels specifically. For more details of these optimization strategies refer to ref. 2.

## SOM 2-2) Model evaluation

The models' performance is evaluated over test data, which is used neither for training the model nor for the validation process. In order to estimate the instantaneous firing rates, the sequences of stimuli presented to the neuron are given to the model according to Eq. 5 (in the Methods), and the spiking history input to the post-spike kernel is simulated, similar to ref. 2. The performance of the model in predicting the neural response is assessed at the level of both firing rate prediction (Supplementary Fig. 4a-c) and also single trial, single spike prediction (Supplementary Fig. 4d-e).

Supplementary figure 4a shows how well the model captures the stimulus-response relationship by evaluating its ability to capture the average firing rate in response to the repeated presentation of a probe in the neuron's RF during the fixation period (500 to 200 ms before saccade) for a sample MT neuron. We then quantify the similarity between actual and model-predicted responses across the population of recorded neurons using the explained variance (EV) measure[4]. Supplementary figure 4b shows that the EV between the model-predicted firing rate and the empirical firing rate (y-axis) matches that obtained between 1000 pairs of average firing rate sequences measured over randomly selected subset of probe-aligned spike trains, used as a measure of inherent variability in the neural data itself (x-axis). The average firing rate sequences were computed by binning the probe-aligned spike response using nonoverlapping windows of 30 ms and smoothing the binned response with a Gaussian window of 5 ms (full width half max) and normalizing to have a mean of zero and unit standard deviation. The model-data EV and data-data EV are highly correlated, showing that the model-predicted response captured the stimulus-response

relationship in the data across the population of the neurons (data-data EV: 84.79±0.54, model-data EV: 79.33 ± 0.78, Pearson correlation: 0.85, $p<0.001$).

Next, we generalized this firing rate level accuracy analysis by evaluating how well the model predicted the firing rate in response to the presentation of experimental sequences of probe stimuli appearing at random locations during the fixation period (500 to 200 ms before saccade). Supplementary figure 4c shows that the correlation coefficient (CC) between the model-predicted firing rate and the empirical firing rate in response to the repeated presentation of a sequence of probe stimuli falls within the level of the inherent trial-by-trial variability. The data-data correlation coefficient was measured between binned firing rates in response to the same 300 ms stimulus sequence; data were randomly split (60%-40%) 15 times and the mean is reported. The binning, smoothing, and normalizing of these data were the same as in the EV analysis. The normalized correlation in percent is calculated as the ratio between the model-data correlation (y-axis) and the data-data correlation (x-axis) and is shown as the diagonal histogram in Supplementary figure 4c. The data-data and model-data CC are positively correlated (data-data CC= 0.46± 0.005 and for the model-data CC it is 0.32± 0.006, Pearson correlation= 0.91, $p<0.001$) showing that the model is able to capture the trial-by-trial variability in the data where exactly same sequence of stimuli were presented to the neuron.

The results show that the model follows the neural response at the single trial level as shown in Supplementary figure 4d. The best and average fit trials in Supplementary figure 4d are chosen based on the largest and median normalized log-likelihood (LL) of trials for each sample neuron. We also analyzed the performance of our model on predicting single spikes and single trials, by assessing how well the model-predicted firing rate matches the

observed spiking data on individual trials. Supplementary figure 4e shows the model's performance in predicting the neural response in the perisaccadic period (from 0 to 150 ms after the saccade onset) versus that measured during fixation (-300 to -150 ms relative to saccade onset) by comparing the normalized log-likelihood of the model prediction in these time periods. The normalized LL, calculated as the LL of the spike trains using the predicted firing rate under the model minus that under a null model and normalized by spike counts, as reported in ref. 2, indicates the amount of information being conveyed by individual spikes and evaluates how closely the model describes the timing of recorded spikes. To calculate the normalized LL, the null model is assumed to be a model where the instantaneous firing rate of the neuron is set to its average firing rate. The distribution of the normalized LL values across the population of neurons shows that the model performance in describing neural data in the perisaccadic period is as good as during the fixation period (perisaccadic normalized LL= 0.17±0.00 bits/sp, fixation normalized LL=0.17±0.00 bits/sp, p=0.75). All reported p-values are from a Wilcoxon signed-rank test unless otherwise stated.

**SOM 3) Calculating the duration of overlap between RF1 and RF2 discriminability**

Supplementary figure 6a,b illustrates how the discriminability at the RF1 and RF2 locations overlap during the perisaccadic period, and how the duration of this overlap time is quantified. The discriminability of the neuron to either RF1 or RF2 ($\gamma_1$and $\gamma_2$) is defined as the maximum discriminability across different delays at each response time (see Methods). The overlap time is then measured as the time where stimuli in both RF1 and RF2 are discriminable. Times $t_1$ and $t_2$ representing the start and end of the overlap time are quantified based on the cumulative sum of the baseline subtracted discriminability traces ($\varphi_1(t) = \int_{t=-50}^{t}(\gamma_1(t) - 0.5)dt$ for RF1 and similarly $\varphi_2(t)$ for RF2). Each cumulative

function $\varphi_1(t)$ and $\varphi_2(t)$ is calculated for -50 to +200 ms relative to the saccade and normalized to their sum over this period. Time $t_1$, marking the start of the overlap time, is then measured as the time relative to the saccade when the normalized $\varphi_1(t)$ passes 70% of its maximum. Similarly, time $t_2$, marking the end of the overlap time, is measured as the time relative to the saccade when the normalized $\varphi_2(t)$ reaches 30% of its maximum. The overlap time is the difference between these start and end times. After removing the integration-relevant STUs the mean population overlap time decreases, and in fact becomes negative, indicating that discriminability around RF1 disappears before discriminability around RF2 arises: a gap in discriminability. The same maps, with and without modulated STUs, are shown separately for V4 and MT neurons in Supplementary figure 5g-h.

**SOM 4) Detectability and discriminability in recorded neural data**

Figure 2c illustrates the detectability and discriminability maps based on the prediction of the model (see Methods for more details). We also calculated the detectability and discriminability maps based on the recorded neural responses. Supplementary figure 5e-f illustrates the detectability and discriminability maps calculated based on the recorded neural responses and averaged over the population of neurons. To calculate the detectability of a probe at an arbitrary time relative to the saccade ($t$) and at a specific delay ($\tau$), the spike count of the neuron is averaged over a 10 ms window around time $t$, in the trials that the probe was presented in a 10 ms window around time $t - \tau$. The detectability is then measured as the AUC of the ROC between the spike count in the trials where the probe was presented versus trials where it was not. Similarly, for the discriminability, we evaluate the ability of the neuron's response at time $t$ to differentiate a probe from its surrounding probes

in terms of the spike count of the neuron in a 10 ms window around time $t$ in the trials that probes were presented at in a 10 ms window around $t - \tau$.

**SOM 5) Future field remapping**

It has been shown that in several sensory areas of the cortex, neurons become responsive to their future field (FF, same as RF2) prior to a saccade (in LIP[5], FEF[6], V2, V3a[7], and V4[8]). In area MT, predictive future field remapping of stable stimuli has not been observed[9], but memory remapping of brief FF stimuli has been reported in MT[10] and MST[11]. Our results are consistent with the memory-remapping phenomena previously reported in MT and V4, with neurons responding to brief probes presented in their FF prior to saccade onset. Supplementary figure 7a illustrates the population neural response to the visual probes presented in the FF of the neurons in the perisaccadic period (-15 to 0 ms to the saccade onset for the MT neurons and -40 to -10 ms to the saccade onset for the V4 neurons) compared to the probe presentations during fixation (fix1 and fix2, 500 to 200 ms before or after saccade onset respectively), for MT (n=332) and V4 neurons (n= 291). Our results show that the neurons in both areas V4 and MT become sensitive to probes which appear in their future field close to the onset of the saccade. To quantify the FF remapping across the population of the neurons, the future field modulation index (FMI) is defined as:

$$FMI = (r_2 - r_1)/(r_2 + r_1) \quad (11)$$

where $r_2$ is the average firing rate in the late response window (70 to 110 ms to the probe onset for the MT neurons and 65 to 130 ms for the V4 neurons) for the probes appearing in the perisaccadic period and $r_1$ is the average firing rate of the neurons to the probes presented at the same location on the screen during the first fixation averaged over the same response

window. For both the V4 and MT neuronal populations, the modulation index is significantly greater than zero ($FMI_{MT}$ = 0.02±0.01, $p=0.001$; $FMI_{V4}$ = 0.06±0.01, $p<0.001$; Supplementary Fig. 7a).

It is important to rule out residual luminance of the FF probe after the eyes have landed as a cause of the response to the perisaccadic FF probes. This "phosphor persistence" was determined to be the basis for some previously reported remapping phenomena[12, 13]. In our case, the sustained residual luminance from a pre-saccadic FF probe would need to last through the duration of the saccade execution to explain the observed responses; this is unlikely given the luminance offset time course of the screen measured with the photodiode (Supplementary Fig. 1b). Nevertheless, to provide additional assurance that residual luminance is not responsible for the observed FF remapping, in a different set of experiments, V4 neurons were tested with both white probes on a black background and black probes on a white background (80% contrast). As shown in Supplementary figure 7c, for 107 V4 neurons both black and white probes show an FF modulation index significantly greater than zero (white probes: 0.1±0.01, $p<0.001$, black probes: 0.06±0.01, $p=0.001$), demonstrating that residual luminance does not account for the FF remapping phenomenon. The FMI is calculated for the black probes presented -20 to +10 ms relative to the saccade onset and in the response window of 65 to 105 ms relative to the saccade onset. The FF modulation index for white and black probes are positively correlated across neurons (Pearson correlation, $r = 0.31$, $p<0.001$), consistent with remapping based on spatial rather than luminance properties of the stimulus. Thus, both the photodiode-measured time-course of stimulus offset and the presence of remapping for black probes on a white background

indicate that phosphor persistence is not responsible for the observed FF remapping phenomenon.

FF modulation was slightly weaker in area MT than in V4 ($FMI_{MT}$ = 0.02±0.01, $FMI_{V4}$ = 0.06±0.01; p=0.09, 291 V4 and 332 MT neurons, rank-sum test). Considering that FF remapping was observed in V4 in both black and white conditions we can rule out the possibility of monitor luminance persistence. While significant FF remapping was observed for the white probes in area MT, there was not significant FF remapping for the black probes (white probes: 0.04±0.01, p=0.007, black probes: -0.03±0.02, p=0.3, Δ white vs. black: 0.08±0.01, p=0.004, signrank test over 33 MT neurons). Thus, FF remapping in MT may depend more on the luminance of the scene and statistics of the stimuli. The finding that in MT, FF remapping depended on the overall luminance and statistics of the scene may also help explain differing levels of FF remapping phenomena observed in MT across studies[9,10].

We also considered the possibility of a role of persistent phosphor luminance in generating the late response enhancement phenomenon. This possibility is slim since the late response enhancement is evoked by the presence of a probe in the RF1 right before the saccade, so even if there was phosphor persistence it would be removed from the RF of the neuron by the subsequent saccade. However, as an additional control we confirmed the presence of the late response enhancement for the black probes presented on the white background ($PMI_{V4}$ = 0.05±0.02, p=0.001 for 107 V4 neurons).

**SOM 6) Timing of the perisaccadic modulation in areas MT and V4**

Supplementary figure 8a-b compares the response map (firing rate as a function of probe position) for a sample V4 (Supplementary Fig. 8a) and MT (Supplementary Fig. 8b)

neuron in the early response window (V4 50 to 60 ms and MT 50 to 70 ms relative to the probe onset) versus the late window (V4 60 to 130 ms and MT 80 to 90 ms relative to the probe onset) in the fixation (500 to 200 ms before the saccade onset) and perisaccadic (-10 to +3 ms relative to the saccade onset) periods. The response map in the late window of the perisaccadic period shows the late response enhancement phenomenon in both areas. To calculate the significance of the perisaccadic modulation of RF1 responses over different response windows, the firing rates in response to the RF1 probe in the perisaccadic period were compared to those in the fixation period using a t-test. Supplementary figure 8c plots the significance of this comparison (t-score) for a sliding 30 ms response window relative to the probe onset. Positive t-score values correspond to an enhancement in the perisaccadic firing rate and the negative values indicate a suppression in the perisaccadic firing rates relative to fixation. As shown in Supplementary figure 8c, perisaccadic probe-evoked responses are initially suppressed and then enhanced (relative to fixation). To separately characterize the timecourse of the perisaccadic suppression and enhancement across the V4 and MT populations, we evaluated the proportion of neurons whose perisaccadic responses were suppressed or enhanced over time relative to the probe onset (Supplementary Fig. 8d-e). To calculate the proportion of the V4 or MT neurons which show significant suppression or enhancement, the response to the RF probe appearing during the perisaccadic or fixation periods was compared for each neuron in a 40ms sliding window, using a one-sided Wilcoxon rank-sum test. A neuron is considered suppressed when the perisaccadic response is significantly lower than the fixation one ($p<0.01$); similarly, enhancement is defined as the perisaccadic response being significantly greater than the fixation response. The 40 ms window slides (1 ms increments) over the 200 ms after the probe onset to find the time-course of these modulations. As shown in Supplementary figure 8d, for V4 neurons

perisaccadic suppression and enhancement were most prevalent around 55 ms and 80 ms after probe onset, respectively. For MT neurons, the maximum prevalence of neurons showing suppression and enhancement was reached 51ms and 83 ms after probe onset. The total proportion of neurons showing suppression (in any 40 ms window from 0 to 90 ms after probe onset t, Wilcoxon ranksum test $p<0.01$) was not different between V4 and MT neurons (Chi squared test, $p=0.62$). The total proportion of neurons showing enhancement (in any 40 ms window from 60 to 200 ms after probe onset, Wilcoxon ranksum test $p<0.01$) was not different between V4 and MT neurons (Chi squared test, $p=0.09$).

We also looked for a relationship between RF eccentricity (e.g., radial distance from fixation) and perisaccadic suppression or enhancement. Supplementary figure 8e shows how the timing of perisaccadic enhancement and suppression change across neurons with RFs at different eccentricities. As shown in the figure, V4 and MT neurons exhibited different patterns of suppression and enhancement as a function of RF eccentricity. A binary logistic regression (BLR) model was fit to the data to evaluate whether the RF eccentricity affects prevalence and timing of suppression or enhancement across V4 and MT neurons. Presence of suppression or enhancement, for each neuron, was assigned if the response in a sliding 40 ms window was significantly different in the perisaccadic period versus fixation (Wilcoxon rank-sum test, $p<0.01$, any window from 0 to 90 ms after probe onset for suppression and 30 to 160 ms after probe onset for enhancement). In the fitted BLR models, the inverse of the RF eccentricities are the independent variable (predictor). The fitted coefficients of the eccentricity in the BLR model was then evaluated to determine the relationship between RF eccentricity and prevalence of suppression and enhancement. The prevalence of suppression did not differ based on RF eccentricity for V4 neurons (the fitted BLR $p=0.34$, no significant

relationship between eccentricity and prevalence of suppression). However, the prevalence of suppression in MT neurons varied significantly based on eccentricity (the BLR model $p<0.01$, fitted coefficient=4.92, $p<0.01$ indicating decreasing prevalence of suppression as the RF eccentricity increases). Response enhancement was also more prevalent for the neurons with more eccentric RFs, in both MT and V4 (V4: BLR model $p=0.02$ and fitted coefficient=-2.08, $p=0.03$; MT: BLR model $p<0.01$ and fitted coefficient=-5.22, $p<0.01$). The fact that MT suppression and enhancement have opposite relationships to eccentricity suggests that rather than resulting from a generic saccade-induced delay in the response of neurons, the two phenomena are likely to be the result of a saccade-induced change of response that differentially affects neurons across the visual field. Also, it should be noted that whereas the suppression and enhancement phenomena can be differentiated in time and their relationship with RF eccentricity, whether they are generated by two separate sources or they are temporally separable response changes due to a single source is not possible to discern in the current study.

As shown in Supplementary figure 8e, the timing of the enhancement was negatively correlated with the eccentricity in both areas MT and V4. The time of enhancement for each neuron was defined as the time of maximum modulation (measured with PMI, as in Fig. 4d) from the period in which enhancement was significant (measured for a 40 ms sliding window from 30 to 160 ms after the probe onset; Wilcoxon rank-sum test, $p<0.01$). In both V4 and MT, the time of peak enhancement was earlier for larger eccentricity RFs (enhancement time and eccentricity correlation V4: $r=-0.14$, $p=0.04$ and MT: $r=-0.21$, $p<0.01$; Pearson correlation).

**SOM 7) Perisaccadic modulation of response in congruent versus incongruent saccades**

As shown in figure 4e, the PMI is significantly greater than zero for the neurons in area MT regardless of the direction of the saccade. The congruent saccades are defined as those in which saccade-induced retinal motion is within 180 degrees of the neuron's preferred motion direction (incongruent saccades induce motion >+90 or <-90 degrees from the neuron's preferred direction of motion). In figure 4e, we show that MT neurons' perisaccadic responses are modulated for saccades that are either congruent or incongruent with the neuron's preferred motion direction. In Supplementary figure 9, we show the perisaccadic change in response for a subset of MT neurons (n =61) which were recorded for both congruent and incongruent saccade directions.

Supplementary figure 9a shows the population average response of 61 MT neurons to an RF probe during the perisaccadic and fixation periods, for congruent and incongruent conditions. As shown, the perisaccadic visual responses are enhanced (compared to fixation) for both congruent and incongruent conditions. Differences later in the response are likely attributable to the saccade target being closer to the RF in the incongruent condition (distance between RF and ST: congruent=19.31±0.56 dva, incongruent=9.28±0.50 dva). The scatter plot of PMI values (Supplementary Fig. 9b) shows that the perisaccadic enhancement for this population of 61 MT neurons is not different for congruent saccades versus incongruent ones, within the same time window used in figure 4 (75-105ms after probe onset). The PMI values for both congruent and incongruent saccades are significantly greater than zero (congruent PMI=0.04±0.02, p=0.024, incongruent PMI=0.07±0.02, p=0.005), and the median PMI is not different between the two saccade directions (congruent versus

incongruent, $p=0.273$). PMI values of individual neurons for congruent vs. incongruent saccades are positively correlated (correlation=0.48, $p<0.001$).

**SOM 8) Saccade target remapping**

Whereas the early component of probe-aligned response mostly reflects the classic RF of the neurons, we found that around the time of saccade the late component of response shows sensitivity to other parts of visual space. As shown in Supplementary figure 7, some neurons become sensitive to RF2 probes in the perisaccadic period (FF remapping). Some neurons show late response enhancement (Fig. 4), a late component of the response to perisaccadic RF1 probes. A late component of the response is also observed for perisaccadic probes around the ST (ST remapping). The ST remapping, FF remapping, and late response enhancement phenomena are observed in varying combinations across the neuronal population. Supplementary figure 10a shows the response pattern for 3 sample neurons, which become sensitive to ST probes in the perisaccadic period (in combination with either late response enhancement or FF remapping), and compares it to the pre- and post- saccadic RFs. The late component of the response is shown for the perisaccadic period in Supplementary figure 10a, illustrating how ST remapping occurs at the same time as FF remapping or late response enhancement. The pre-(left) and post-(middle) saccadic RFs of the neuron are calculated by averaging the response to probes presented more than 100 ms before or after the saccade onset across a 30 ms window around the maximum response time relative to the probe onset. For the perisaccadic maps the firing rate of the neurons are averaged for the probes presented -10 to +3, -10 to -3, and -10 to -3 ms relative to saccade onset and the response windows are 105-115, 65-90, and 80-90 ms relative to probe onset.

To further verify that the FF modulation also exists independent of the ST modulation, we measured the FMI for the neurons where the FF of the neuron is far from the ST (distance between FF probe and ST > 8 dva). The results show that the FF remapping exists for these neurons in both MT and V4 areas ($FMI_{V4}$= 0.06±0.01, p=0.001, n=93, $FMI_{MT}$=0.06±0.01, p<0.001, n=216), confirming that FF remapping (far from the ST) exists in this population.

Supplementary figure 10b-d shows the detectability map for ST probes across the time to saccade for a sample neuron (Supplementary Fig. 10b), the population of neurons (Supplementary Fig. 10c), and versus the RF1 and RF2 detectability (Supplementary Fig. 10d). Supplementary figure 10b shows the detectability map for a sample ST probe for a sample neuron with ST remapping. To measure the population detectability maps in Supplementary figure 10c-d, we first identify the probe locations near the saccade target that do not overlap with RF1 or RF2, or the vector connecting them. More specifically, first we select all the probes around the saccade target if they are within a window of 50% of the saccade vector centered at the ST of each neuron. Then, to avoid any confound with the conventional RF or the corresponding retinal locations during the eye movement, probes are discarded if they are closer than 2 dva to any point along the vector connecting the RF1 probe to the RF2. Next we determined which of these probes near the ST are evoking significantly different responses during the perisaccadic vs. fixation periods. We first used an ANOVA test on the difference of the firing rate of the neurons in the late window of the perisaccadic versus average of the fixation period before the saccade in response to all ST probes. If the responses across all the ST probes are modulated perisaccadically for a given neuron (p<0.01, ANOVA), we then use a post hoc Bonferroni test to identify the modulated probes

($p<0.05$). Plots in Supplementary figure 10c,d are averaged across modulated probes across the population of 51 V4 and MT neurons with significant modulation at an individual ST probe location. The ST discriminability contour shows where the detectability map crosses threshold of 0.55. Supplementary figure 10d shows detectability at the RF1, RF2, and ST locations, across the time of probe and response relative to the saccade.

Supplementary figure 10e shows the co-occurrence of the late response enhancement, FF remapping, and ST remapping phenomena in the population of neurons. Neurons were only included in the analysis if the neuron's FF was far from the ST (corresponding to RF eccentricity >4 dva), to avoid potentially confounding FF and ST modulations (n = 553 V4 and MT neurons). Significant RF and FF modulations in individual neurons were defined as described previously, based on a comparison of perisaccadic and fixation firing rates at the RF or FF probe locations (Wilcoxon rank-sum test, $p<0.01$). ST modulations were tested for near-ST probes (as described for Fig. S10c-d), using an ANOVA for perisaccadic vs. fixation responses (criteria $p<0.01$).

We evaluated if the late response enhancement was more likely to occur in neurons which also displayed FF or ST remapping. The proportion of neurons showing late response enhancement was not different for neurons with vs. without ST remapping (Chi squared test MT: $p=0.25$, n=317; V4: $p = 0.21$, n=236). The proportion of neurons with late response enhancement was not different for neurons with vs. without FF remapping (Chi squared test, MT: $p=0.54$, n=332; V4: $p=0.74$, n=291). Thus late response enhancement appears to be independent of the previously reported FF and ST remapping phenomena.

**Supplementary Figures 1-10**.

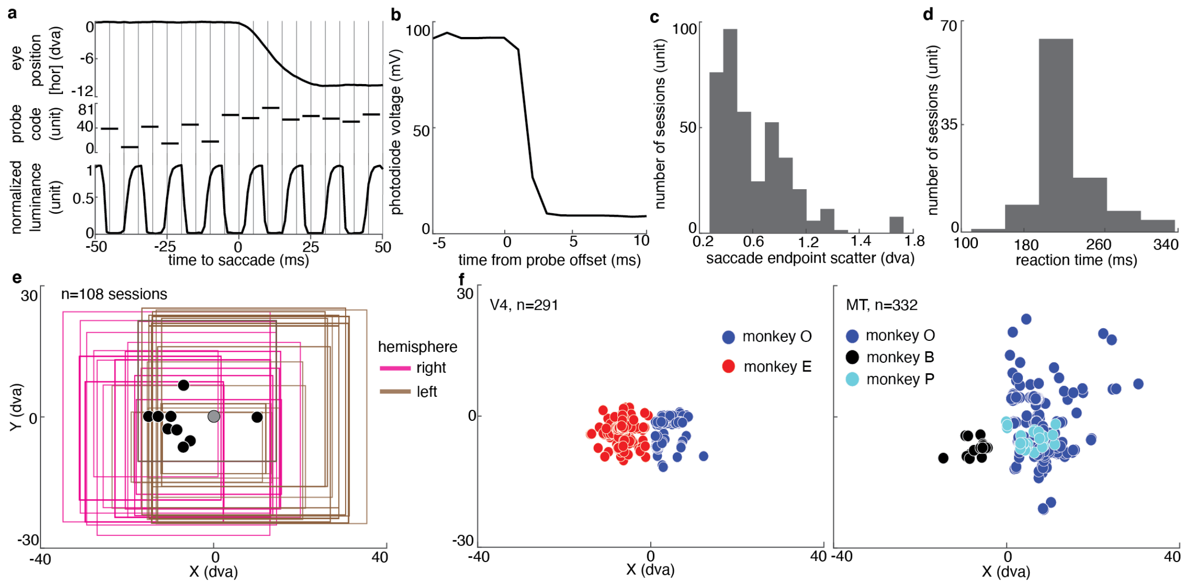


**Supplementary Figure 1. Details of visual stimuli, behavior, and RFs. a,** Eye position (top), visual probe locations (middle), and corresponding photodiode voltage over time for a sample trial, aligned to the time of saccade onset. A separate stimulus driving the photodiode is toggled (on/off) simultaneous with the onset of each visual probe. **b,** Luminance decay of monitor, measured with a photodiode, aligned to stimulus offset (mean across 810 trials of a sample session, error bars too small to be visible). **c,** Distribution of mean saccade endpoint scatter across 108 recording sessions. **d,** Distribution of monkeys' mean reaction time (time between go cue and saccade onset) across 108 recording sessions. **e,** Spatial extent and positioning of the probe grids and saccade targets across 108 sessions. Outline colors reflect grid positioning for recordings from right (pink) and left (brown) hemispheres. Black dots represent the saccade target positions and the gray dot marks the

FP. **f,** RF center locations for the population of V4 (left) and MT neurons (right); colors indicate the animal. Source data are provided as a Source Data file.

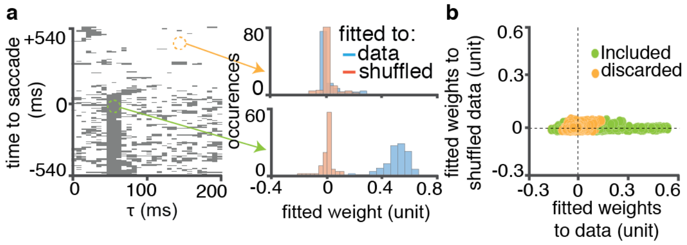


**Supplementary Figure 2. Dimensionality reduction procedure for STU selection. a,** The gray area represents the temporal distribution of STUs with a significant stimulus-response relationship (gray) for a probe inside RF1 of a sample neuron. The STUs for the RF1 probe occur primarily at delays around the latency of the sample neuron (50-70 ms, x-axis) and for probes appearing before saccade onset (-540 to 0 ms, y-axis). After the saccade, prevalence of the STUs for RF1 probes diminishes, showing that the eye movement has moved this probe out of neuron's RF. The distribution of fitted weights for different subsets of trials is shown for sample STUs with non-significant (orange) and significant (green) stimulus-response relationships. The histograms show the distribution of fitted weights of a simplified single STU estimated using a linear-nonlinear-Poisson model across different random subsets of trials (n = 100 random selections) versus the fitted control weights where trials are randomly shuffled. **b,** Mean estimated weights for each STU of RF1 of the sample neuron in A, fitted to the data (x-axis) and shuffled (y-axis; mean of 100 random subsets). STU locations with non-significant stimulus response relationships are shown in orange while the rest of the STUs are shown in green. Source data are provided as a Source Data file.

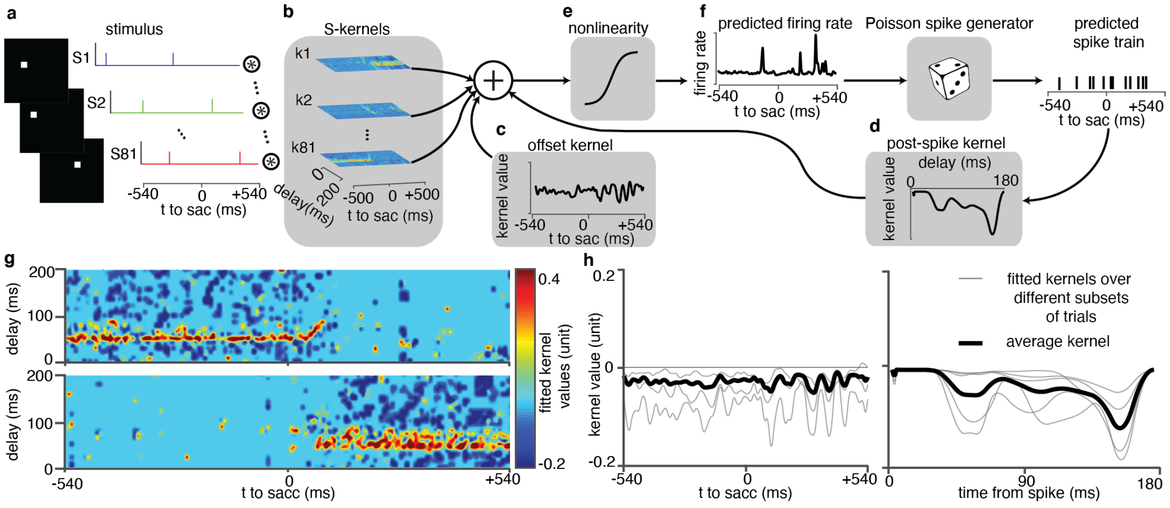


**Supplementary Figure 3. Schematic of sparse variable generalized linear model structure and fitted components. a,** The input to the model is shown by a set of stimulus sequences along each spatial dimension for 3 sample locations. **b,** S-kernels representing the neuron's sensitivity at different times. **c,** The offset kernel representing the changes in the neuron's baseline activity across time relative to the saccade. **d,** Post-spike kernel representing neuron's inherent features like refractoriness or burstiness. **e,** The nonlinear response function. The input to the model gets convolved with the S-kernels and is then summed with the offset kernel and the feedback signal through the post-spike kernel. The resulting signal is then passed through the sigmoidal nonlinearity to generate a predicted instantaneous firing rate of the neuron. **f,** The predicted firing rate is then passed through a conditionally Poisson generator to generate the predicted spike train. **g,** The fitted S-kernel for a sample neuron for RF1 (top) and RF2 (bottom) locations. **h,** The fitted offset kernel (left) and post-spike kernel (right) for the sample neuron. The gray traces show the fitted kernel to the randomly chosen subsets of the data (65% of the data).

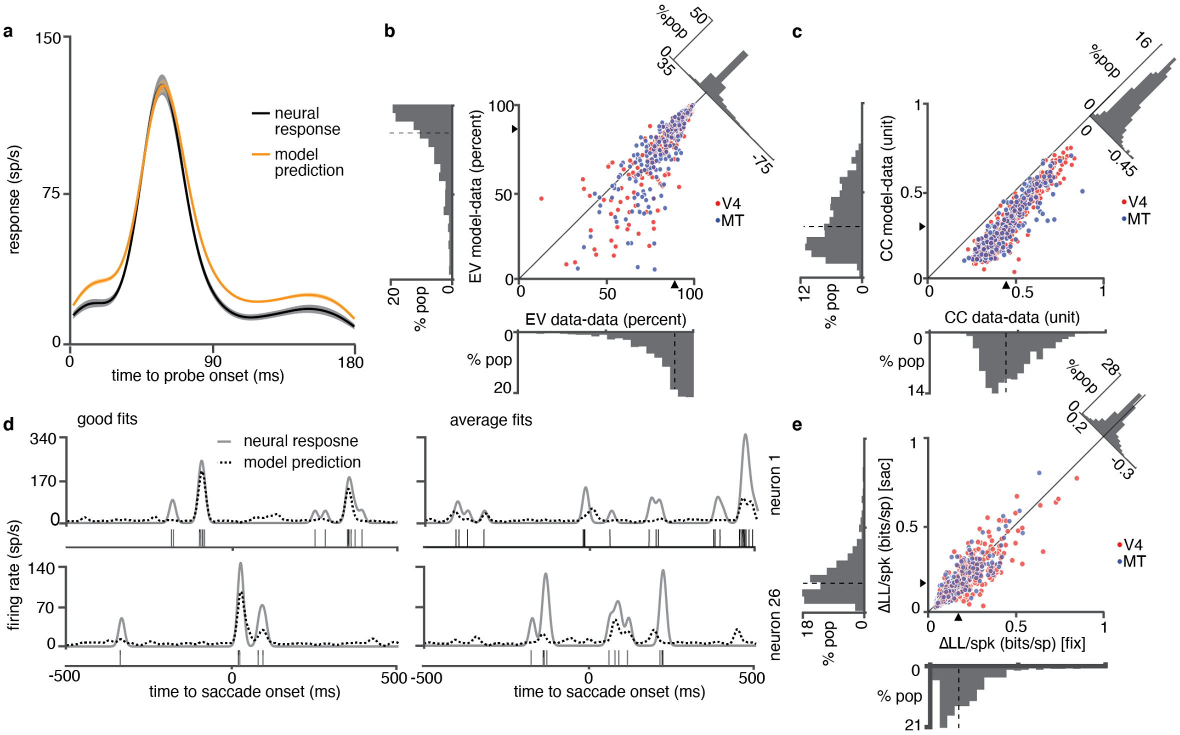


**Supplementary Figure 4. Performance of the model in predicting the neural responses.** **a,** Black and orange traces show the stimulus aligned neural response (mean±SEM) and the corresponding prediction of the model, for a sample MT neuron. The response of the neuron is averaged over repetitions of the probe presentations inside the neuron's RF in the fixation period (500 to 200 ms before the saccade onset) and smoothed with a Gaussian window of 20 ms (full width half max). The error bars are calculated over repetitions of the probe across trials. **b,** The percentage of the explained variance (EV) for the model-predicted firing rate for all neurons in area MT and V4 (n = 623), for the stimulus-aligned neural response when a stimulus is presented inside the RF during the fixation period. In **b,c** and **e** the black triangles indicate medians, blue and red colors represent MT and V4 neurons respectively, and histograms show the marginal distributions (left, bottom) and the difference (upper-right). **c,** The normalized cross-correlation (CC) between the model-predicted response and the data, compared to the data-data correlation, averaged over different sequences of stimuli

in all conditions for the population of 623 MT and V4 neurons. **d,** The traces show the model-predicted firing rate (dashed line) versus the recorded neural response (solid line) for sample trials which were of good fit (left; best LL) and average fit (right, median LL) for two sample neurons (top and bottom). Spikes in each trial shown as black vertical lines below the smoothed traces. **e,** Comparison of the normalized LL of the recorded spiking response under the model predicted firing rate in the perisaccadic versus fixation period. Source data are provided as a Source Data file.

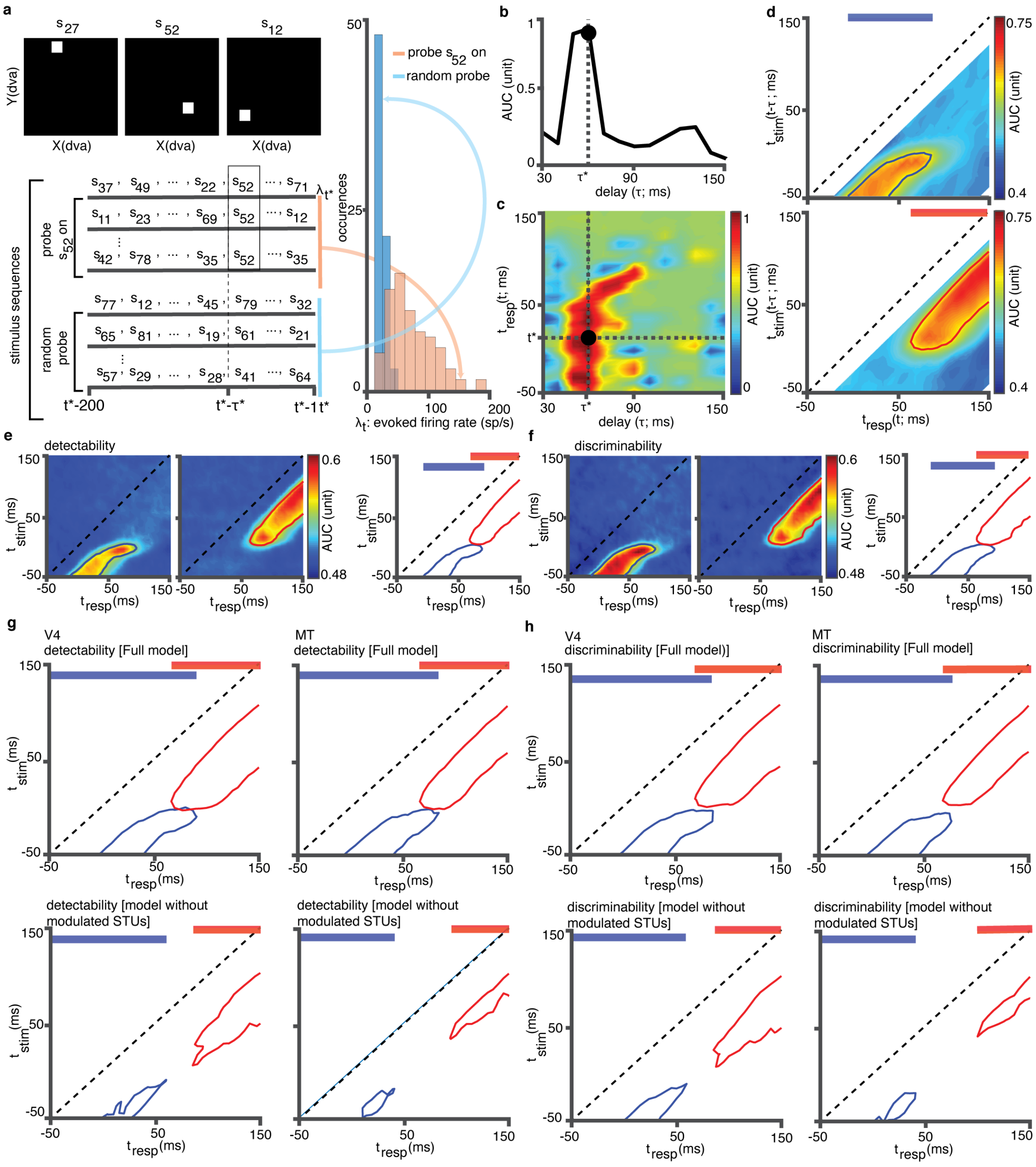

**Supplementary Figure 5. Detectability across delay and time to saccade. a,** Illustration of the method for calculating the detectability of a sample probe $s_{52}$ at a specific time to saccade ($t^*$) and delay relative to probe presentation ($\tau^*$). Top: White squares show the location of three example visual probes, for probes $s_{27}$ , $s_{52}$ , $s_{12}$ where $s_1, \dots s_{81}$ represent the 81 possible locations on the 9 by 9 stimulus grid. Middle: Example stimulus sequences,

in which either sample probe $s_{52}$ appears at a specific delay relative to $t^*$ (top three rows), or a random probe appears at the same delay relative to $t^*$ (bottom three rows). Bottom: Histograms show the firing rate distributions for the trials in which sample probe $s_{52}$ appears at a specific delay relative to $t^*$ (red), or a random probe appears at the same delay (blue). The AUC of an ROC between these two distributions for each time and delay value produces the detectability values shown in **b-d**. **b,** Detectability of an example MT neuron across delay values for one time to saccade ($t^*$ = +10 ms). Detectability peaks at the response latency of the neuron ($\tau^*$ = 60 ms). **c,** Detectability for the same example neuron shown in **b**, across both delay and time to saccade. Horizontal dotted line corresponds to the $t^*$ plotted in **b**; vertical dotted line corresponds to delay with maximum detectability. **d,** Detectability for the same neuron shown in **b** and **c,** plotted as a function of time between stimulus and saccade (y-axis) and response relative to saccade (x-axis), for probe locations in RF1 (left) and RF2 (right). Outlines show the period when the AUC > 0.61. The projected lines on top show the times that the detectability threshold was exceeded for RF1 (blue) and RF2 (red). **e,** Average detectability map over the population of all V4 and MT neurons measured based on the recorded neural responses. **f,** Average discriminability map over the population of all V4 and MT neurons measured based on the recorded neural responses. **g,** Model-based readout of detectability (as in Fig. 2c&e), for the full model (top) or the model without modulated STUs (bottom), separately for V4 (left) and MT (right) neurons. **h,** Model-based readout of discriminability (as in Fig. 2c&e), for the full model (top) or the model without modulated STUs (bottom), separately for V4 (left) and MT (right) neurons. Overlapping red and blue bars indicate an integrated readout and nonoverlapping cases indicate a gap in the readout. Source data are provided as a Source Data file.

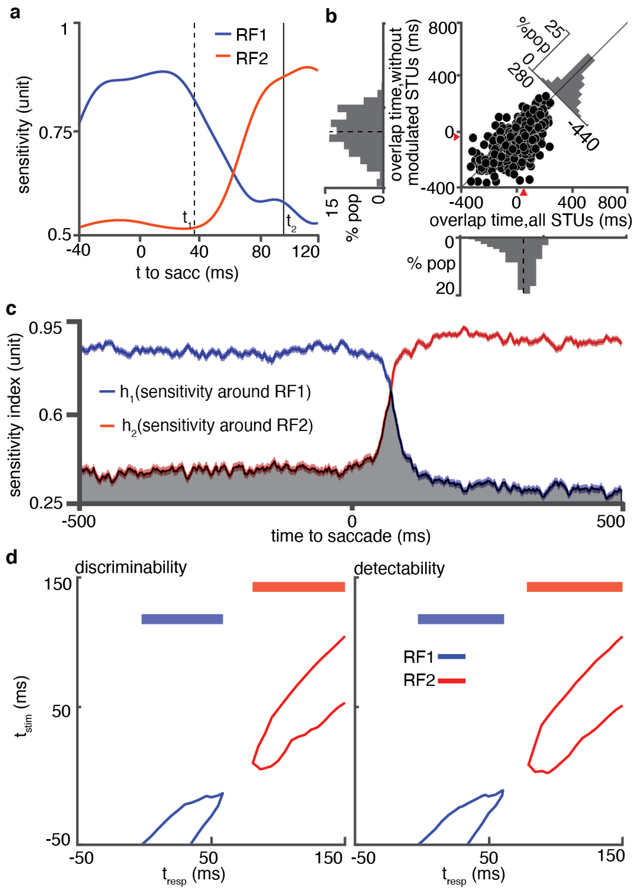


**Supplementary Figure 6. Identifying the overlap time with and without the modulated STUs and determining the integration-relevant STUs. a,** Discriminability metric of a sample neuron around RF1 (blue) and RF2 (red) over time relative to the saccade. Time $t_1$ (vertical dashed line) and $t_2$ (vertical solid line) represent the start and the end of the overlap time. **b,** Overlap time across the population of the neurons with and without the modulated STUs. The red triangles and the dashed lines indicate medians; histograms show the marginal distributions (left, bottom) and the difference (upper-right). **c,** Sensitivity index of the neuron to probes around RF1 (blue) and RF2 (red) over time relative to the saccade. The sensitivity of the neuron to the RF1 drops after the saccade while the sensitivity to the probes around RF2 increases. The combined sensitivity, measured as the shared area under the curve of both RF1 and RF2 sensitivity, is shown in gray. If eliminating an STU resulted in a decrease

in the combined sensitivity, it was deemed integration-relevant. **d,** Effect of eliminating integration-relevant STUs on the discriminability (left) and detectability (right) for the neuronal population. Contour lines show time periods in which discriminability (left) and detectability (right) at the RF1 (blue) and RF2 (red) locations exceeded the same threshold used in figure 2. Projections on top show a gap in discriminability/detectability during the perisaccadic period. Source data are provided as a Source Data file.

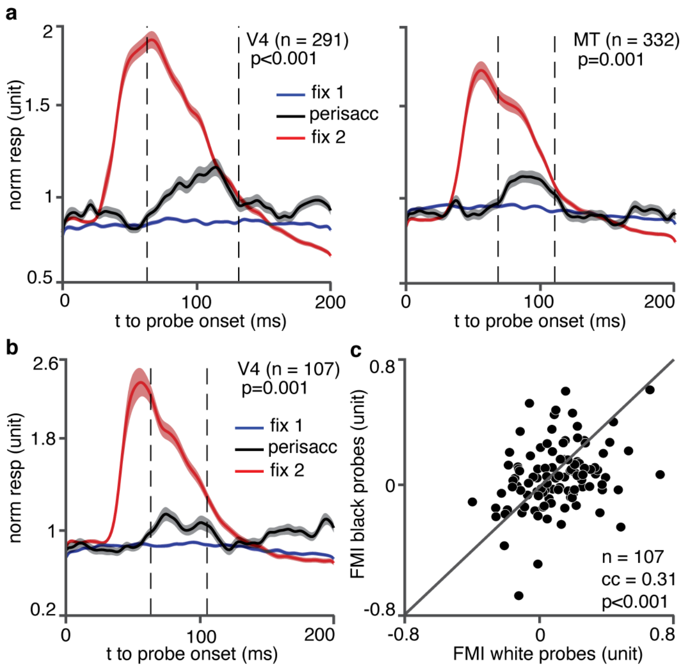


**Supplementary Figure 7. Future field remapping in areas V4 and MT. a,** The probe aligned response in area V4 (left) and MT (right), for the white probes presented in RF2 during the pre-, peri-, and post-saccadic periods (blue, black, and red respectively). The dashed lines indicate the response window used for the FMI calculation ($p_{V4} = 1.01 * 10^{-8}$). **b,** The probe aligned response in area V4 for the black probes presented in RF2 during the pre-, peri-, and post-saccadic periods (blue, black, and red respectively). Plots are mean±SEM across neurons; p-values in **a-b** are for Wilcoxon signed-rank tests of FMI values in the time window between the dashed lines. **c,** FMI for the black probes on the white background versus the white probes on the black background over 107 V4 neurons ($p_{correlation} = 9.68 * 10^{-4}$). ‘n=107’ for **b-c** indicates number of neurons for which both the black-probe-on-white-background and the white-probe-on-black-background paradigms were recorded within the same session. The correlation coefficient and p-value in **c** are for a Pearson correlation. Source data are provided as a Source Data file.

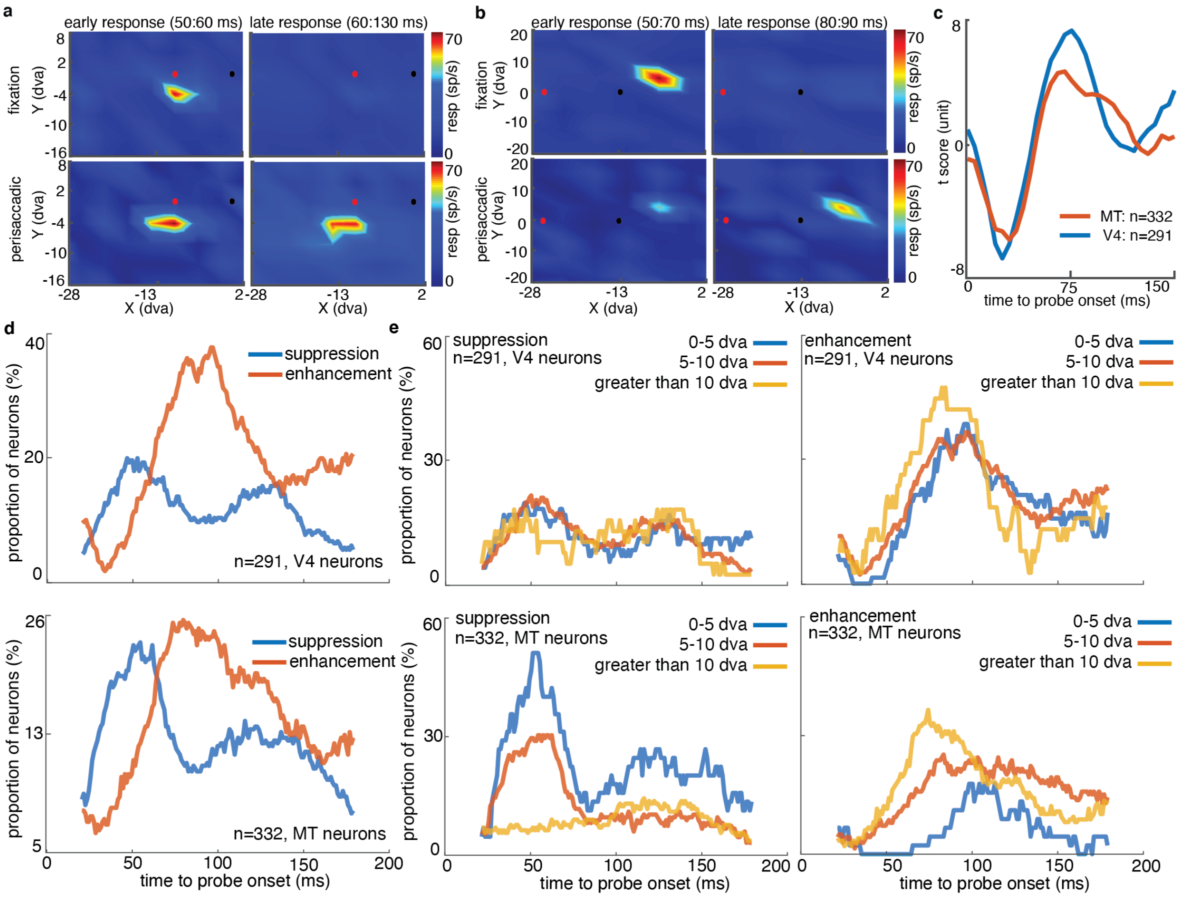


**Supplementary Figure 8. Response suppression and enhancement over time for V4 and MT neurons.** Response map (firing rate in response to probes at various locations) of a sample **a,** V4 and **b,** MT neuron, for the early (left) and late (right) response windows for probes in the fixation (top) and perisaccadic (bottom) periods. **c,** Plot shows the timing of the perisaccadic modulation (V4: blue and MT: red) measured as the t-score of the average firing rate in response to the RF1 probe in the perisaccadic period versus the fixation period, for different response windows relative to the probe onset. The x-axis value indicates the start of the response window relative to the probe onset (window duration 30 ms). **d,** Proportion of neurons in area V4 (top) and MT (bottom) with significant suppression (blue) and enhancement (orange) in a 40 ms sliding window. **e,** Proportion of V4 (top) and MT

(bottom) neurons with significant suppression (left) and enhancement (right) over time relative to probe onset, for RFs of various eccentricities (colors).

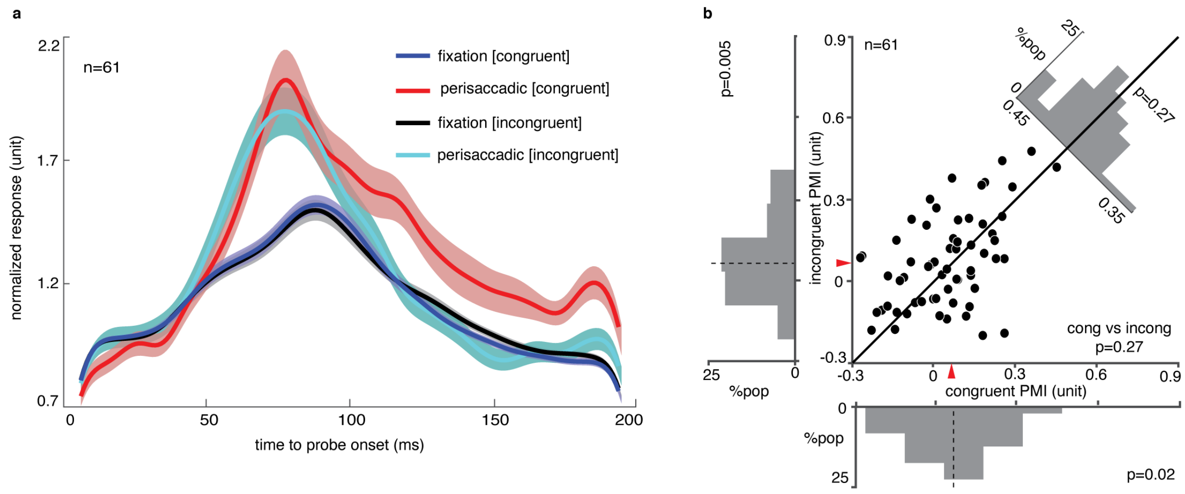


**Supplementary Figure 9. Saccade direction does not alter the strength of the late response to perisaccadic RF1 stimuli in MT. a,** Average response (mean±SEM) of 61 MT neurons to the RF probe in the perisaccadic period (red, cyan) and fixation period (blue, black), for congruent (red, blue) and incongruent (cyan, black) saccade directions. **b,** The scatter plot shows the PMI for the saccades congruent versus incongruent with the preferred motion direction of 61 MT neurons. Each data point shows the PMI of a single neuron for saccades in two opposite directions. The histograms along the x and y axis show the distribution of PMI values for congruent and incongruent saccades, respectively; red arrows mark the median of each marginal distribution. The upper right histogram shows the distribution of the difference in PMI for congruent versus incongruent saccades. 'n=61' indicates the number of neurons for which saccades in two opposite directions were recorded within the same session. The p-values on the marginal distributions are for Wilcoxon rank sum tests; the one on the scatter plot and upper right histogram is for a Wilcoxon signed-rank test. Source data are provided as a Source Data file.

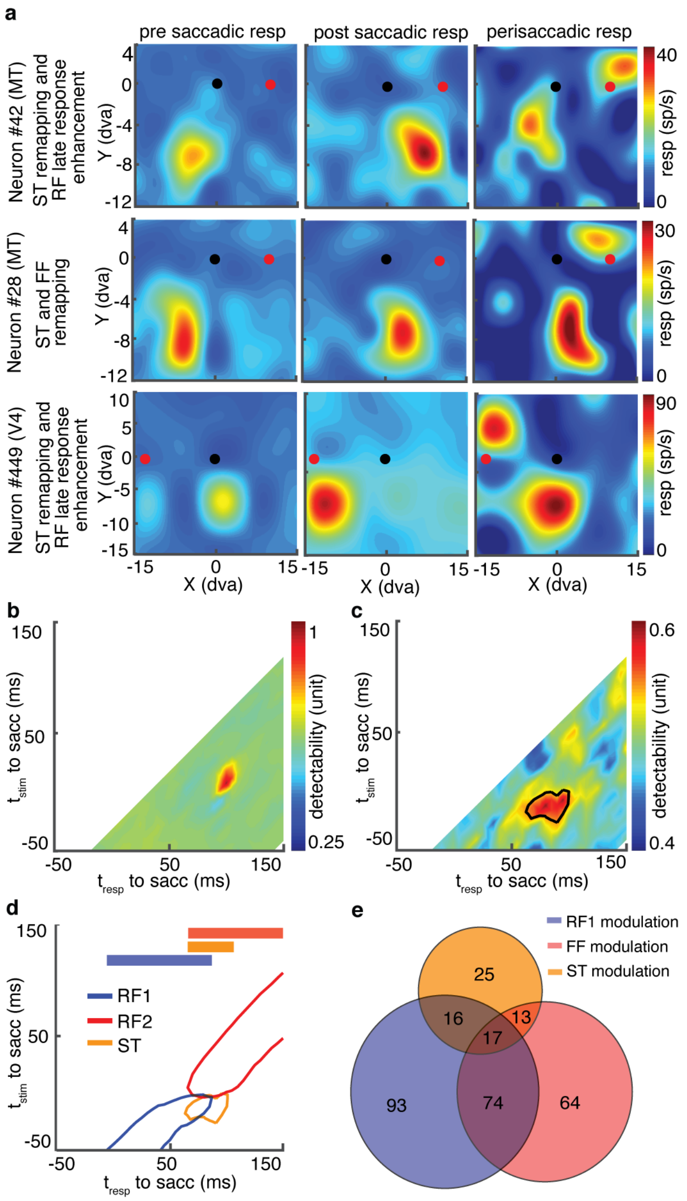


**Supplementary Figure 10. Coexistence of RF, FF, and ST modulations. a,** Three sample neurons (top to bottom) responding to perisaccadic probes around the ST location. Heat maps show the response to probes at different locations before (left), after (middle), and around (right) the saccade onset. The fixation point (black) and saccade target (red) are shown as filled circles. Top and bottom neurons show ST remapping and RF late response enhancement; middle neuron shows ST and FF remapping. **b,** Detectability of a probe around the ST, for a sample neuron, as a function of time between saccade and response (x-axis) or

probe onset (y-axis). **c,** Detectability of probes around the ST for the population of 51 neurons with significant modulation at an individual near-ST probe location, as a function of time between saccade and response (x-axis) or probe onset (y-axis). The contour is showing where the detectability exceeds a threshold. **d,** The detectability of the RF1, RF2, and ST probes for the population. Lines on top show at what times relative to the saccade the detectability exceeds a threshold for RF1, RF2, and ST. **e,** Venn diagram showing the number of neurons exhibiting perisaccadic modulation at the RF, FF, and ST probe locations. Low-eccentricity neurons were excluded in order to differentiate ST and FF activity. 297 out of 553 neurons show modulation at one or more of these locations.

## References:


1. Hwang J, Mitz AR, Murray EA. NIMH MonkeyLogic: Behavioral control and data acquisition in MATLAB. Journal of neuroscience methods 323, 13-21 (2019).

2. Akbarian A, Niknam K, Parsa M, Clark K, Noudoost B, Nategh N. Developing a Nonstationary Computational Framework with Application to Modeling Dynamic Modulations in Neural Spiking Responses. IEEE Transactions on Biomedical Engineering, (2017).

3. Niknam K, Akbarian A, Clark K, Zamani Y, Noudoost B, Nategh N. Characterizing and dissociating multiple time-varying modulatory computations influencing neuronal activity. PLoS computational biology 15, e1007275 (2019).

4. Schoppe O, Harper NS, Willmore BD, King AJ, Schnupp JW. Measuring the performance of neural models. Frontiers in computational neuroscience 10, 10 (2016).

5. Colby C, Goldberg M. The updating of the representation of visual space in parietal cortex by intended eye movements. Science 255, 90-92 (1992).

6. Sommer MA, Wurtz RH. Influence of the thalamus on spatial visual processing in frontal cortex. Nature 444, 374 (2006).

7. Nakamura K, Colby CL. Updating of the visual representation in monkey striate and extrastriate cortex during saccades. Proceedings of the National Academy of Sciences 99, 4026-4031 (2002).

8. Neupane S, Guitton D, Pack CC. Two distinct types of remapping in primate cortical area V4. Nature communications 7, 10402 (2016).

9. Ong WS, Bisley JW. A lack of anticipatory remapping of retinotopic receptive fields in the middle temporal area. The Journal of neuroscience 31, 10432-10436 (2011).

10. Yao T, Treue S, Krishna BS. An attention-sensitive memory trace in macaque MT following saccadic eye movements. PLoS biology 14, e1002390 (2016).

11. Inaba N, Kawano K. Eye position effects on the remapped memory trace of visual motion in cortical area MST. Scientific reports 6, 22013 (2016).

12. Jonides J, Irwin DE, Yantis S. Integrating visual information from successive fixations. Science 215, 192-194 (1982).

13. Jonides J, Irwin DE, Yantis S. Failure to integrate information from successive fixations. Science,  (1983).
