# Supplementary material for: A sensory memory to preserve visual representations across eye movements
Source: Nat Commun. 2021 Nov 8;12:6449. doi: 10.1038/s41467-021-26756-0 (PMC8575989; doi:10.1038/s41467-021-26756-0)
Supplement: Supplementary file 2 — Reporting Summary [file 41467_2021_26756_MOESM2_ESM.pdf]

nature research

Corresponding author(s): Behrad Noudoost and Neda Nategh

Last updated by author(s): Apr 30, 2021

Reporting Summary

Nature Research wishes to improve the reproducibility of the work that we publish. This form provides structure for consistency and transparency in reporting. For further information on Nature Research policies, see our Editorial Policies and the Editorial Policy Checklist.

Statistics

For all statistical analyses, confirm that the following items are present in the figure legend, table legend, main text, or Methods section.

n/a	Confirmed	
☐	☒	The exact sample size (n) for each experimental group/condition, given as a discrete number and unit of measurement
☐	☒	A statement on whether measurements were taken from distinct samples or whether the same sample was measured repeatedly
☐	☒	The statistical test(s) used AND whether they are one- or two-sided<br>*Only common tests should be described solely by name; describe more complex techniques in the Methods section.*
☐	☒	A description of all covariates tested
☐	☒	A description of any assumptions or corrections, such as tests of normality and adjustment for multiple comparisons
☐	☒	A full description of the statistical parameters including central tendency (e.g. means) or other basic estimates (e.g. regression coefficient) AND variation (e.g. standard deviation) or associated estimates of uncertainty (e.g. confidence intervals)
☐	☒	For null hypothesis testing, the test statistic (e.g. F, t, r) with confidence intervals, effect sizes, degrees of freedom and P value noted<br>*Give P values as exact values whenever suitable.*
☒	☐	For Bayesian analysis, information on the choice of priors and Markov chain Monte Carlo settings
☒	☐	For hierarchical and complex designs, identification of the appropriate level for tests and full reporting of outcomes
☐	☒	Estimates of effect sizes (e.g. Cohen's d, Pearson's r), indicating how they were calculated

Our web collection on statistics for biologists contains articles on many of the points above.

Software and code

Policy information about availability of computer code

Data collection	The data has been recorded using Cheetah v5 and Central v7 electrophysiology recording softwares. Stimulus presentation and reward delivery was controlled using custom codes in Matlab (2018) MonkeyLogic toolbox.
Data analysis	Plexon Offline Sorter (v3) and Blackrock Offline Spike Sorter (BOSS, v 1.03.00) have been used to sort the spikes. Commercial Matlab (2018) toolboxes from Blackrock and Neuralynx has been used to read data into Matlab software. All data analysis was performed using custom written codes in Matlab.

For manuscripts utilizing custom algorithms or software that are central to the research but not yet described in published literature, software must be made available to editors and reviewers. We strongly encourage code deposition in a community repository (e.g. GitHub). See the Nature Research guidelines for submitting code & software for further information.

Data

Policy information about availability of data

All manuscripts must include a data availability statement. This statement should provide the following information, where applicable:

- Accession codes, unique identifiers, or web links for publicly available datasets
- A list of figures that have associated raw data
- A description of any restrictions on data availability

Sample neurons are avialbale at the GitHub page here: https://github.com/nnategh/SVGLM/tree/master/assets/data . The processed neural data are available from the corresponding author upon request. The data generated in this study are provided in the Source Data file.

April 2020

Field-specific reporting

Please select the one below that is the best fit for your research. If you are not sure, read the appropriate sections before making your selection.

☒ Life sciences ☐ Behavioural & social sciences ☐ Ecological, evolutionary & environmental sciences

For a reference copy of the document with all sections, see nature.com/documents/nr-reporting-summary-flat.pdf

Life sciences study design

All studies must disclose on these points even when the disclosure is negative.

Sample size	In total 623 (291 V4 and 332 MT) neurons were included in the analysis, recorded from 4 macaque monkeys. These sample sizes are typical and sufficient numbers for single unit recording studies (for example Rezayat et. al., 2021, Nat. Comms. and Bichot et. al., 2021, Nat. Comms.)
Data exclusions	From a population of 709 well-isolated neurons, 86 neurons were discarded because they did not respond to any probe stimuli before and/or after the saccade, and the rest were used for analyses.
Replication	291 V4 and 332 MT neurons were recorded across 4 different animals and our results show that the effect exists in the population of neurons across 4 animals.
Randomization	There was no grouping across animals under study. However, the randomization was performed for probe presentation sequence and choice of subset of trials for model fitting and evaluation. In the saccade congruency analysis, the congruent and incongruent saccades are intervened randomly.
Blinding	Blinding is not relevant in this study since there was no grouping.

Reporting for specific materials, systems and methods

We require information from authors about some types of materials, experimental systems and methods used in many studies. Here, indicate whether each material, system or method listed is relevant to your study. If you are not sure if a list item applies to your research, read the appropriate section before selecting a response.

Materials & experimental systems

n/a	Involved in the study	
☒	☐	Antibodies
☒	☐	Eukaryotic cell lines
☒	☐	Palaeontology and archaeology
☐	☒	Animals and other organisms
☒	☐	Human research participants
☒	☐	Clinical data
☒	☐	Dual use research of concern

Methods

n/a	Involved in the study	
☒	☐	ChIP-seq
☒	☐	Flow cytometry
☒	☐	MRI-based neuroimaging

Animals and other organisms

Policy information about studies involving animals; ARRIVE guidelines recommended for reporting animal research

Laboratory animals	4 adult male rhesus macaque monkeys were used in this study (11, 13, 6 and 6 years old).
Wild animals	The study did not involve wild animals
Field-collected samples	The study did not involve samples collected from the field.
Ethics oversight	All experimental procedures complied with the National Institutes of Health Guide for the Care and Use of Laboratory Animals and the Society for Neuroscience Guidelines and Policies. The protocols for all experimental, surgical, and behavioral procedures were approved by Institutional Animal Care and Use Committees of the University of Utah.

Note that full information on the approval of the study protocol must also be provided in the manuscript.
